# Quantitative benefit-risk assessment of methylprednisolone in multiple sclerosis relapses

**DOI:** 10.1186/s12883-015-0450-x

**Published:** 2015-10-16

**Authors:** Ola Caster, I. Ralph Edwards

**Affiliations:** Uppsala Monitoring Centre (UMC), Box 1051, SE-751 40 Uppsala, Sweden; Department of Computer and Systems Sciences, Stockholm University, Postbox 7003, SE-164 07 Kista, Sweden

**Keywords:** Glucocorticoids, Corticosteroids, MS, Neurology, Neuropathy, Demyelinating diseases, Pharmacoepidemiology, Pharmacovigilance, Clinical epidemiology, Decision analysis

## Abstract

**Background:**

High-dose short-term methylprednisolone is the recommended treatment in the management of multiple sclerosis relapses, although it has been suggested that lower doses may be equally effective. Also, glucocorticoids are associated with multiple and often dose-dependent adverse effects. This quantitative benefit-risk assessment compares high- and low-dose methylprednisolone (at least 2000 mg and less than 1000 mg, respectively, during at most 31 days) and a no treatment alternative, with the aim of determining which regimen, if any, is preferable in multiple sclerosis relapses.

**Methods:**

An overall framework of probabilistic decision analysis was applied, combining data from different sources. Effectiveness as well as risk of non-serious adverse effects were estimated from published clinical trials. However, as these trials recorded very few serious adverse effects, risk intervals for the latter were derived from individual case reports together with a range of plausible distributions. Probabilistic modelling driven by logically implied or clinically well motivated qualitative relations was used to derive utility distributions.

**Results:**

Low-dose methylprednisolone was not a supported option in this assessment; there was, however, only limited data available for this treatment alternative. High-dose methylprednisolone and the no treatment alternative interchanged as most preferred, contingent on the risk distributions applied for serious adverse effects, the assumed level of risk aversiveness in the patient population, and the relapse severity.

**Conclusions:**

The data presently available do not support a change of current treatment recommendations. There are strong incentives for further clinical research to reduce the uncertainty surrounding the effectiveness and the risks associated with methylprednisolone in multiple sclerosis relapses; this would enable better informed and more precise treatment recommendations in the future.

**Electronic supplementary material:**

The online version of this article (doi:10.1186/s12883-015-0450-x) contains supplementary material, which is available to authorized users.

## Background

Glucocorticoids are the only pharmacological intervention with a demonstrated effect on multiple sclerosis (MS) relapses, with high-dose short-term methylprednisolone being the currently recommended first line treatment [1]. Nevertheless, the optimal methylprednisolone treatment regimen is unknown [1], and meta-analysis has even suggested that low-dose methylprednisolone may be as efficacious as the high-dose regimen [2]. A whole array of different adverse effects is attributed to glucocorticoids, many of which are dependent on dose and duration of treatment [3, 4]. Recently, high-dose methylprednisolone was associated with hepatotoxicity [5, 6], a previously unrecognised risk that may also warrant consideration. Hence, there is a clear need for a systematic joint evaluation of the beneficial and adverse effects of methylprednisolone in the management of MS relapses, to challenge treatment recommendations, support clinical decision making and inform future research [7]. Specifically, neurologists and MS patients would be well served by a comparison between low- and high-dose methylprednisolone, to maximise chances of treatment benefit while avoiding unnecessary risk of adverse effects.

There are several systematic reviews that investigate the use of methylprednisolone and other glucocorticoids in MS relapse management [1, 2, 8–10]. Although there is a paucity of data from formal studies, some of these reviews contain quantitative analyses with respect to effectiveness. However, experiences of adverse effects are typically presented separately, and to the best of our knowledge there exists no previous evaluation that considers the likelihood and desirability of relevant beneficial and adverse effects jointly.

A number of methods have been proposed for formal benefit-risk assessment [11–13], most of which focus on regulatory decisions regarding initial market approval. However, current regulatory guidelines put clear emphasis also on the benefit-risk balance in the post-marketing setting, and formal assessments are required in the face of significant new risks [14]. We have previously devised a methodology for modelling the utility of drug effects that is appropriate to the post-marketing setting, as it does not require timely and costly elicitation studies [15]. It also avoids the questionable assumptions inherent to methods based on aggregating health state utility over time, e.g. using quality-adjusted life years [15].

The primary aim of this study is to provide a quantitative benefit-risk assessment of methylprednisolone in MS relapse management, to determine whether treatment is to be recommended, and, if so, whether high or low dose is preferable. Our main finding in this respect is that low-dose methylprednisolone is an inferior alternative both to high-dose methylprednisolone and to the no treatment choice, based on available data. The secondary aim is to demonstrate how various methods can be combined through probabilistic decision analysis to yield a transparent and rigorous framework for post-marketing benefit-risk assessment that can accommodate relevant information from disparate sources.

## Methods

### Overview

Drug benefit-risk assessment is here approached as the analysis of a treatment decision problem for a hypothetical representative of the relevant patient population. The same framework could be used for a real patient by incorporating his or her specific preferences.

The flow of the evaluation largely follows that of customary decision analysis [16]: the decision problem, its objective and its alternatives are defined; the relevant effects are identified and modelled in a tree to form clinical outcomes; probability and utility variables are estimated; and each alternative is evaluated with respect to expected utility as a basis for comparison. Expected utility is an overall measure of how preferable an alternative appears.

In addition, the evaluation adopts probabilistic sensitivity analysis [17], meaning that each probability and utility variable is specified as a distribution and sampled, resulting in distributions of the alternatives’ respective expected utilities. The primary evaluation metric is the preference rate, which measures the fraction of sampling iterations in which a given alternative has the highest expected utility [15]. The preference rate of an alternative therefore estimates the probability of that alternative being the preferred one, given the specified model.

**Fig. 1:**
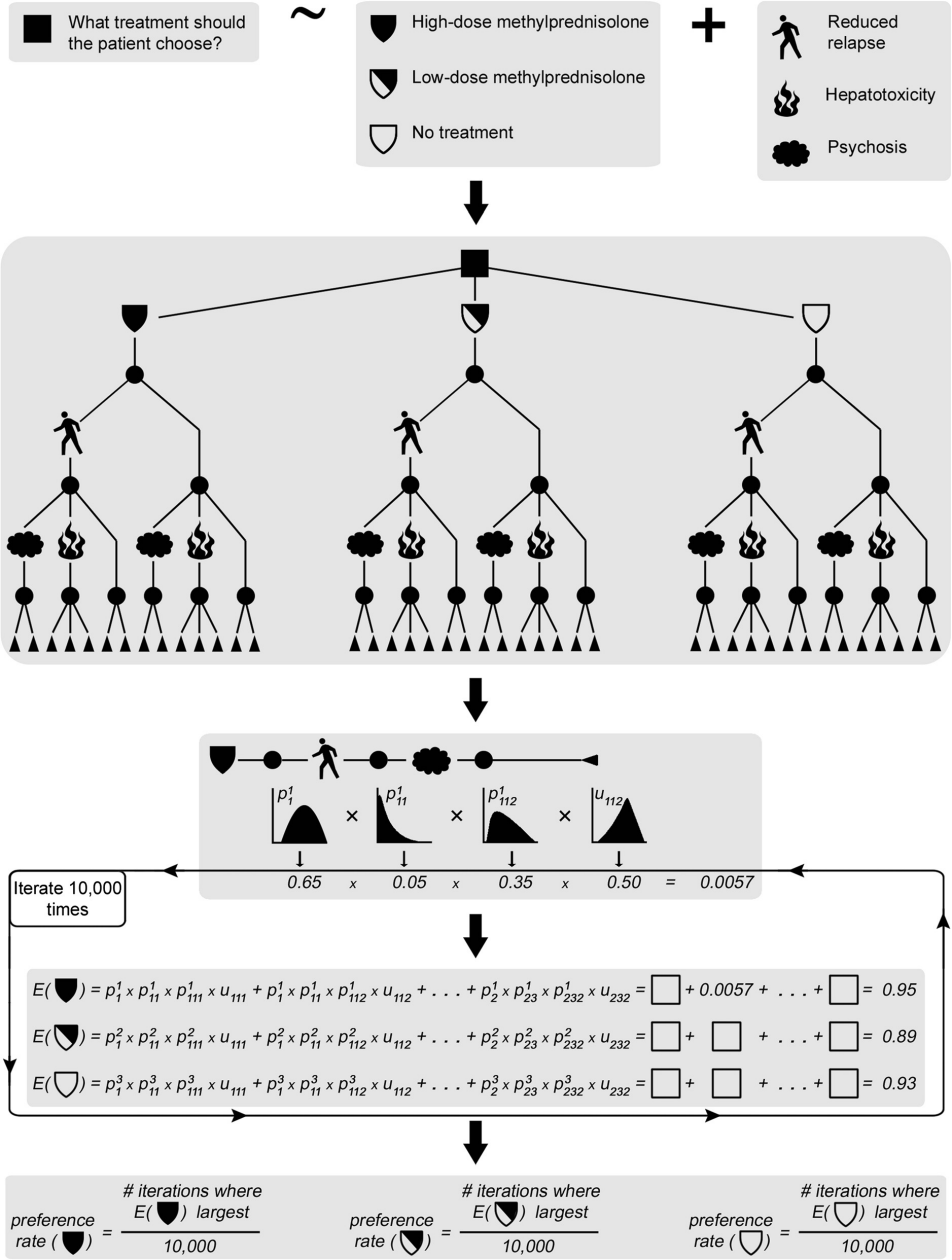
Overview of the applied drug benefit-risk assessment framework. 1st panel: It is identified what decision problem is considered; what the alternatives are; and which effects should be included. 2^nd^ panel: A decision tree model is constructed. 3^rd^ panel: For each clinical outcome, i.e. branch in the tree, distributions are specified for probability and utility variables. These are denoted by *p* and *u*, respectively. Here, one of the clinical outcomes from high-dose methylprednisolone (reduced relapse – psychosis – second unspecified consequence of psychosis) is used for illustration. Superscripts denote alternatives; subscripts denote paths as the tree branches. For each iteration of the probabilistic analysis, values are sampled for all variables, and expected utilities (denoted *E*) are computed for all alternatives, as seen in the 4^th^ panel. The expected utility is the probability-weighted sum of utilities over all clinical outcomes, as indicated in the equations. In this example, the same 14 clinical outcomes are considered for all alternatives. 5^th^ panel: Finally, the preference rate of each alternative is computed. Note that the entire scheme or selected parts thereof could be subjected to (non-probabilistic) sensitivity analysis

This framework is illustrated in Fig. 1, including an explanation of how expected utility is calculated.

### Definition of the decision problem

This assessment analyses a treatment decision of a putative MS patient in acute relapse, with the objective of maximising health during the course of the relapse. Three alternatives are considered: high-dose methylprednisolone, low-dose methylprednisolone and the no treatment choice. High dose was defined as at least 2000 mg methylprednisolone cumulatively during at most 31 days, and low dose was defined as less than 1000 mg cumulatively during the same period of time. The time horizon of the assessment is the duration of a single relapse, which was taken to be 6 months [18]. Optic neuritis is here considered a different indication than MS relapses and hence excluded from the assessment. No differentiation is made with respect to the route of administration.

### Selection of beneficial and adverse effects

The most common clinical endpoint in controlled trials of MS relapses is an improvement of at least one point on the expanded disability status scale (EDSS) [19]. Hence this degree of improvement was adopted as our definition of benefit. It was labelled a ‘reduced relapse’, in contrast to a ‘standard relapse’ where there is less or no improvement.

Serious and non-serious adverse effects were handled differently in the analysis. The latter were considered jointly as a group, because their main significance from a benefit-risk perspective is likely to be their aggregated burden as a nuisance to patients.

Serious adverse effects were defined as being manifested by either life-threatening or persistently disabling reactions. These effects were selected from VigiBase®, the WHO international database of suspected adverse drug reactions [20], since this data source reflects actual concerns about drug treatment in clinical practice and captures rare events unlikely to be seen in small clinical trials. All reports in VigiBase as of May 2012 listing methylprednisolone were extracted, and those reports where treatment could be classified as high- or low-dose were retained as two groups. (For details on the dose calculations, see Additional file 1). A frequency listing was constructed of reported MedDRA Preferred Terms and High-Level Terms, for the two groups separately. A clinical reviewer (IRE) went through the lists separately, and each encountered term that was considered potentially life-threatening or persistently disabling, and reasonably likely to be due to treatment, was mapped to a preliminary term grouping. The top ten adverse effects thus constructed for each dose group were then taken further and rigorously defined as groups of MedDRA Preferred Terms. During the review, the actual frequencies of the various reported terms were hidden.

For each included adverse effect, three different serious outcomes were considered: death, persistent disability and life-threatening though non-lethal reactions. While a lethal outcome is relatively straightforward to capture, the other two outcomes were identified either intrinsically by the nature of the reported term, or based on explicit information on the reports. (For complete definitions, see Additional file 2). Within a given report, the outcome classification of an adverse effect was hierarchical in the order listed above. This means that, for example, if two reactions on the same report suggested hepatotoxicity, of which one reaction was persistently disabling and the other life-threatening, the report would be counted only towards the persistent disability outcome. However, different reactions signifying separate adverse effects on the same report were counted separately and were therefore not necessarily coupled with the same outcomes. Only adverse effect-outcome combinations reported at least three times for both groups together were further considered.

### Modelling of beneficial and adverse effects

All considered effects were modelled together in a tree structure. The small illustrative decision tree in the second panel of Fig. 1 can be used to view the general modelling strategy. The top level corresponds to the three alternatives, each of which is followed by the same sub-tree. This sub-tree, in turn, contains three levels, where the first corresponds to the beneficial effect. The second level contains the serious adverse effects, assumed for simplicity to be mutually exclusive on account of their rarity. Finally, the third level either corresponds to the outcome of the serious effect from the second level (psychosis or hepatotoxicity in the figure); or, in case of no serious adverse effect, the third level delineates two possible events: no adverse effect at all, or at least one non-serious adverse effect. Each branch thus constructed forms one possible clinical outcome.

### Estimation of probability variables

As illustrated in the third panel of Fig. 1, each clinical outcome entails a series of events that each has an associated probability variable with a distribution. In the example used in Fig. 1, these events are in turn reduced relapse, psychosis and some unspecified serious outcome of psychosis. In general, estimation of three types of probability variables is required for each treatment alternative: the effectiveness, i.e. the probability of a reduced relapse; the risk of any non-serious adverse effect; and the respective risks of the included serious adverse effect-outcome combinations.

#### Effectiveness

Data to estimate the effectiveness of the various alternatives was taken from published clinical trials. All papers included in, cited by, or citing any of the available systematic reviews on methylprednisolone in MS were considered [1, 2, 8–10, 21, 22]. Study arms where patients were given either high- or low-dose methylprednisolone as defined above or placebo for at most 31 days were included from trials fulfilling the following criteria:

- Included patients were in acute relapse and diagnosed with either relapsing-remitting or progressive MS.
- The trial was randomised and treatment was blinded to both patients and clinical assessors.
- Patients were assessed clinically, with results reported as the fraction of patients with an improvement of at least one EDSS point compared to start of treatment, or an equivalent thereof.

If several EDSS assessments were made in a single trial, the latest within the interval between 14 and 28 days from start of treatment was used.

The respective effectiveness distributions for the considered alternatives were then estimated by combing the fractions of improved patients reported in the various identified studies, using the hierarchical beta-binomial model with a non-informative prior distribution [23]. Sampling from the posterior distributions relied on Markov chain Monte Carlo (MCMC) simulation with the Metropolis-Hastings algorithm [24, 25]. (For details, see Additional file 3).

#### Risk of any non-serious adverse effect

Data to estimate the risk of one or more occurrences of non-serious adverse effects were also taken from published clinical trials. The same basic search strategy as described for the effectiveness data was used, but treatment arms were included on other criteria, namely:

- The trial was prospective, but not necessarily randomised or blinded.
- Adverse events were reported in such a way that the number of affected patients could be inferred.

Risk distributions were estimated in the same way as for effectiveness, with the exception of low-dose methylprednisolone. The reason was insufficient data: only two trials were identified [26, 27], each with only ten patients on low-dose methylprednisolone and a statement that no adverse events were observed. Instead, it was assumed that the risk for low-dose methylprednisolone should lie between the risk for placebo and that for high-dose methylprednisolone; therefore it was uniformly sampled from the intervals formed by the posterior draws for those two alternatives.

#### Risk of serious adverse effects

The limited number of clinical trials performed for methylprednisolone in MS relapses, in combination with their small sample sizes, makes this source of evidence insufficient to quantify the risks of serious adverse effects: for high-dose methylprednisolone, only two events in total for all included serious adverse effects were reported across the identified trials. Similarly, no published observational studies on methylprednisolone or other glucocorticoids in association with these adverse effects could be used for risk quantification: these studies either used different treatment definitions (e.g. with respect to dose or duration), different outcome definitions, or else they were not designed to estimate risk as per-alternative probabilities, which is required in decision analysis.

Instead a novel approach was used, in which upper limits on true population risks are calculated as reporting ratios in collections of individual case reports [28]. Such risk limits were computed for the included serious adverse effect-outcome combinations from within VigiBase. The reporting ratio denominators included all available reports, whether methylprednisolone was listed as suspected (S), interacting (I), or concomitant (C). The numerators included all S and I reports, while only those C reports were included that did not contain information implicating another drug. Also, for the numerators a requirement was set that the time from drug initiation to onset of the reaction should be at most 180 days. This methodology is further detailed in Additional file 4, with a proper account of the underlying assumptions.

To maintain a probabilistic analysis, different plausible distributions were assigned the various risks over the intervals from zero to their respective upper limits [28]. (For details, see Section ‘Sensitivity analyses’).

It should be noted that the method depicted here deviates slightly from the illustration in Fig. 1: sampling is for the probability of a serious adverse effect-outcome combination directly, not separately for the effect and the outcome. However, this difference is not influential as the total probability for the adverse effect is simply the sum of those for the various outcomes. The conditional probability of a specific considered outcome is then the fraction of the total probability contributed from that particular outcome.

Because no limits could be computed for the no treatment alternative, it was assumed that some proportion of the risk from active treatment could be classified as background risk that would apply to the no treatment alternative as well. This background risk was calculated, for each adverse effect-outcome combination, as the average between the sampled values for low- and high-dose methylprednisolone, respectively, multiplied by the proportion. Different values were imputed for this unknown proportion; see Section ‘Sensitivity analyses’.

### Estimation of utility variables

As illustrated in the fourth panel of Fig. 1, the sampled probability values are combined with sampled utility values in the expected utility calculations. Here, a tailored approach was used to sample from the utility variables of the respective clinical outcomes [15, 29]. In this approach, each utility is first assigned a standard uniform distribution, and qualitative relations are specified that relate the desirability of the various clinical outcomes to each other. Then, the totality of these relations is used to shift the initial distributions accordingly. It is also possible to specify minimum differences between utility variables in case sufficient separation has not been achieved. (For details, see Additional file 5). The main benefit of this approach is that external data are not required; in particular, timely and costly elicitation studies can be avoided.

A clinical expert (IRE) performed the qualitative modelling, blinded to any estimates of probability variables. Because this benefit-risk assessment is made for the whole patient population rather than a specific patient, only logically implied or clinically well motivated relations were used. As recommended [15], a minimum utility difference was included between non-lethal and lethal outcomes, to reflect their intrinsically different nature. Modelling was performed separately for patients starting their relapse at EDSS 4 and EDSS 5, respectively, to investigate whether relapse severity has any influence on the overall benefit-risk profile.

### Sensitivity analyses

Four unknown components of the assessment were altered in a series of sensitivity analysis scenarios. Two of these components concern the risk of serious adverse effects, and two concern the sampling from utility variables.

**Fig. 2:**
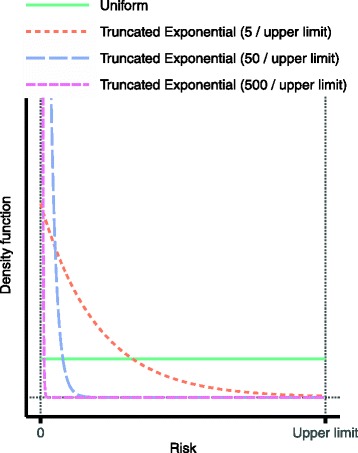
Probability distributions used over the derived risk intervals for serious adverse effects. The uniform distribution puts equal belief over the entire interval from zero to the upper limit, and so has an expected value of half the upper limit. The truncated exponential distributions, on the other hand, put increasingly more belief on low risks. Their expected values are 0.19, 0.020 and 0.0020 times the upper limit, respectively. Note that to benefit the clarity of the display, the graphs for the two left-most exponential distributions have been truncated: in reality they extend much higher for risks close to zero

As mentioned, different types of distributions over the derived risk intervals for the serious adverse effects were investigated; these are shown in Fig. 2. Further, the proportion of the sampled risk values that is attributed to the background, and that therefore determines the values for the no treatment alternative, was varied between 0 and 50 %.

The minimum utility difference between non-lethal and lethal outcomes was altered over the range from 0 to 0.99. Also, as mentioned, different sets of qualitative utility relations were used for patients at different levels of relapse severity.

In addition, a set of auxiliary sensitivity analyses were undertaken to determine the extent to which different variables contributed to the overall uncertainty. This was done by replacing all sampled values for a given variable by the median of the sampled values for that variable.

As depicted in Fig. 1, the probabilistic sensitivity analysis within each investigated scenario was based on 10,000 sampling iterations, yielding one preference rate for each alternative. All sampled values for all probability and utility variables in all scenarios, as well as the resulting expected utilities and preference rates, are freely available; for details, see ‘Availability of supporting data’.

## Results

### Included serious adverse effects

A total of eleven serious adverse effects were included, as shown in Table 1; hence there was a considerable overlap among the ten adverse effects chosen from the two dose groups. The respective definitions of these adverse effects are given in Additional file 6. In total, 26 serious adverse effect-outcome combinations were sufficiently often reported to be considered in the study.

**Table 1:** Serious adverse effects included into the benefit-risk assessment, listed alphabetically

Adverse effect	Included outcomes		
	Death	Persistent disability	Life-threatening reaction
Acute severe allergy	Yes	No	Yes
Cardio-pulmonary distress	Yes	Yes	Yes
Diabetes	Yes	Yes	Yes
Gastrointestinal haemorrhage	Yes	Yes	Yes
Hepatotoxicity	Yes	Yes	Yes
Myopathy	No	Yes	Yes
Osteonecrosis	No	Yes	No
Pancreatitis	Yes	No	No
Psychosis	No	Yes	Yes
Seizure	Yes	Yes	Yes
Ventricular arrhythmia/cardiac arrest	Yes	Yes	Yes

With the exception of hepatotoxicity, all of the included adverse effects are labelled for methylprednisolone [30]. However, an association between high-dose methylprednisolone and hepatotoxicity has recently been reported, with strong support for a causal link [5, 6]. At the same time, the list does not contain some of the commonly discussed adverse effects of glucocorticoids, such as skin reactions, eye reactions and infections [3, 4].

### Structural model

**Fig. 3:**
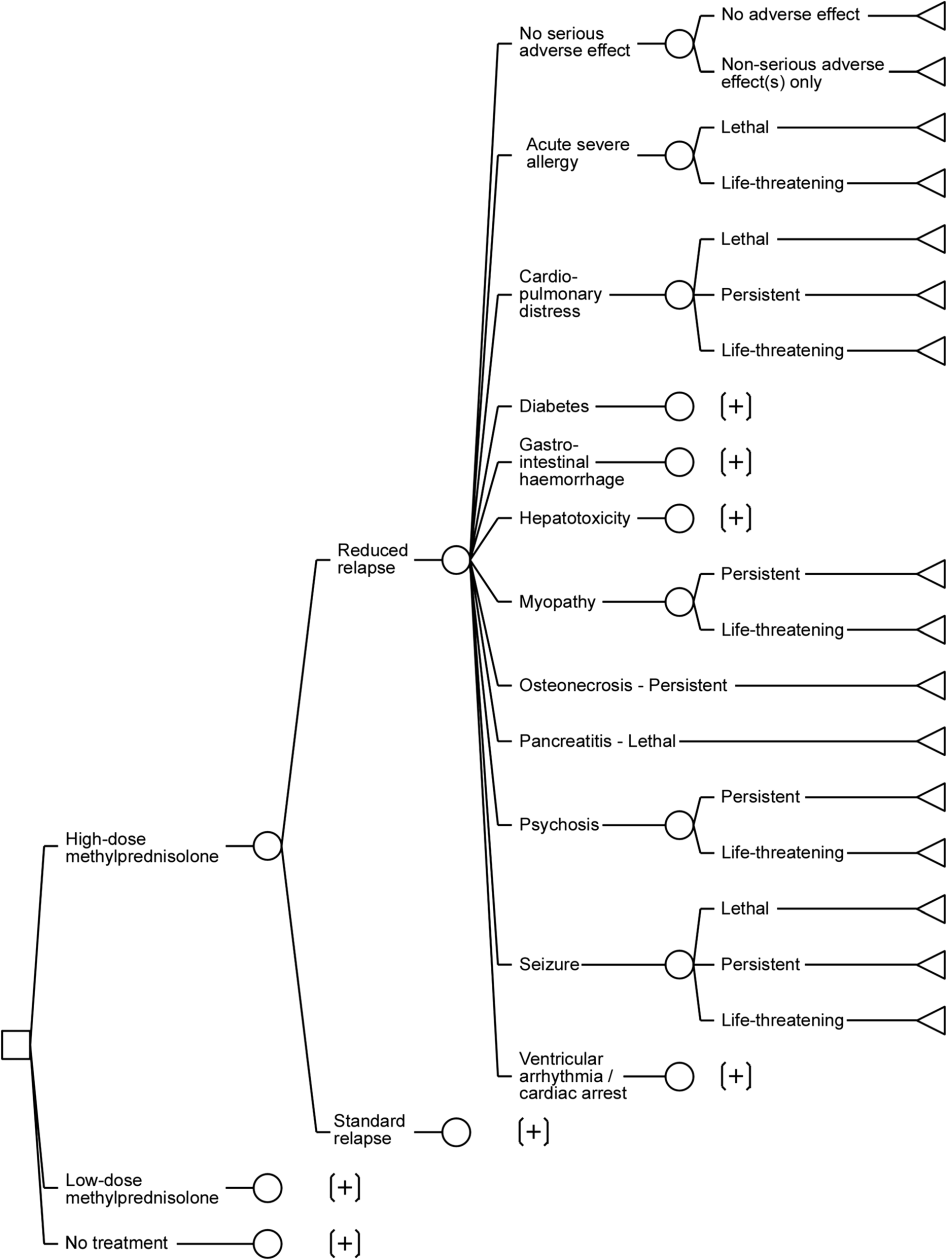
Decision tree model used for the benefit-risk assessment of methylprednisolone in MS relapses. Any sub-tree denoted with [+] is identical to the sub-tree immediately above it

The decision tree used for the evaluation is depicted in Fig. 3. In total there are 56 clinical outcomes considered for the three alternatives.

### Effectiveness

The study arms included for the estimation of effectiveness are reported in Table 2. (For details concerning the article selection process, see Additional file 7). There are eight, three and five arms included for high-dose methylprednisolone, low-dose methylprednisolone and placebo, respectively. These include in total 152, 62 and 156 patients, respectively, from ten different studies [26, 27, 31–38]. The included patients’ EDSS scores at start of treatment are centred between 4.0 and 5.0 for a majority of studies.

**Table 2:** Details of included study arms for the estimation of effectiveness

Intervention	Study	Cumulative dose	Route	Duration	Day of assessment	Fraction with reduced relapse	Relapse severity (baseline EDSS)	Diagnostic criteria
High-dose methylprednisolone	Durelli 1986^a^ [31]	7035 mg^b^	IV	15 days	15	10/11	5.8 (mean)	Poser 1983 [51]
	Milligan 1987 [32]	2500 mg	IV	5 days	28	10/13	4.0 (median)	McDonald 1977 [52]
	La Mantia 1994 [27]	5750 mg	IV	14 days	14	8/10	4.6 (mean)	McDonald 1977 [52]
	Barnes 1997 [33]	3000 mg	IV	3 days	28	13/38	6.0 (median)	Not stated
	Sellebjerg 1998 [34]	3676 mg	PO	15 days	21	14/26	4.5 (median)	Poser 1983 [51]
	Visser 2004 [35]	2500 mg^c^	IV	5 days	28	6/9	3.5 (median)	Not stated
	Ramo-Tello 2013 [36]	3000 mg	IV	3 days	28	15/23	4.0 (median)	McDonald 2005 [53]
	Ramo-Tello 2013 [36]	3750 mg	PO	3 days	28	15/22	3.0 (median)	McDonald 2005 [53]
Low-dose methylprednisolone	Milanese 1989 [26]	390 mg	IV	14 days	30^d^	3/10	4.9 (mean)	McDonald 1977 [52]
	La Mantia 1994 [27]	390 mg	IV	14 days	14	6/10	4.7 (mean)	McDonald 1977 [52]
	Barnes 1997 [33]	588 mg	PO	21 days	28	20/42	5.0 (median)	Not stated
Placebo	Miller 1961 [37]	-	IM	21 days	21	4/18^e^	Not stated	Not stated
	Rose 1970 [38]	-	IM	14 days	28	39.25/94^f^	5.2 (mean)	Rose 1968 [54]
	Durelli 1986^a^ [31]	-	IV	15 days	15	4/10	5.9 (mean)	Poser 1983 [51]
	Milligan 1987 [32]	-	IV	5 days	28	2/9	4.0 (median)	McDonald 1977 [52]
	Sellebjerg 1998 [34]	-	PO	15 days	21	6/25	4.0 (median)	Poser 1983 [51]

*IV* intravenous, *PO* per oral, *IM* intramuscular

^a^ Only the 15 days controlled period of this trial is considered here

^b^ Based on a weight of 70 kg

^c^ Patients also received 2 % human albumin

^d^ This study lacked reported assessments in the 14–28 day interval

^e^ No EDSS measurement performed; the result refers to the fraction with an ‘undoubted response to treatment’

^f^ Conversion based on the assumption that a DSS of e.g. 4 is equally likely to correspond to EDSS 4.0 as EDSS 4.5

**Fig. 4:**
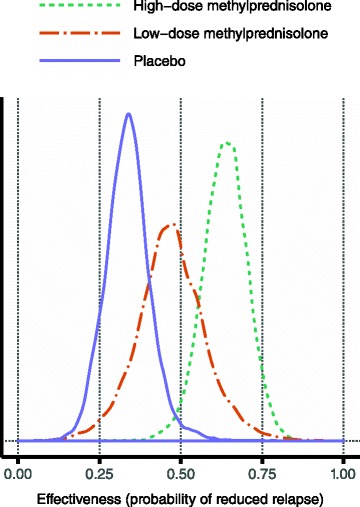
Estimated effectiveness for the three alternatives. The posterior median values were 0.64, 0.47 and 0.34 for high-dose methylprednisolone, low-dose methylprednisolone and placebo, respectively

The estimated distributions for effectiveness, i.e. the probability of having a reduced relapse, are displayed in Fig. 4. Although the distributions are wide, the ordering of the alternatives is the one that would be pharmacologically expected, and the one depicted from the crude fractions in Table 2.

### Risk of non-serious adverse effects

Table 3 lists the study arms included for the estimation of the risk of non-serious adverse effects, taken from ten different studies [27, 31, 34, 36, 38–43]. (For a detailed flow of the article selection process, see Additional file 7). For high-dose methylprednisolone there are eleven arms including a total of 301 patients, and for placebo there are three arms with 129 patients in total. For both treatments there is clearly great heterogeneity across the different studies.

**Table 3:** Details of included study arms for the estimation of risk for non-serious adverse effects

Intervention	Study	Cumulative dose	Route	Duration	Follow-up	Fraction with at least one adverse event
High-dose methylprednisolone	Abbruzzese 1983 [39]	8400 mg^a^	IV	15 days	Not stated	3/30^b^
	Durelli 1986^c^ [31]	7035 mg^a^	IV	15 days	15 days	9.2/13^d^
	Thompson 1989 [40]	3000 mg	IV	3 days	84 days	1/29
	Sellebjerg 1998 [34]	3676 mg	PO	15 days	56 days	23/26
	La Mantia 1994 [27]	5750 mg	IV	14 days	14 days	0/10
	Soelberg-Sorensen 2004 [41]	3000 mg^e^	IV	3 days	182 days	30/40^f^
	Martinelli 2009 [42]	5000 mg	IV	5 days	28 days	11/20
	Martinelli 2009 [42]	5000 mg	PO	5 days	28 days	15/20
	Ramo-Tello 2013 [36]	3000 mg	IV	3 days	28 days	24/24
	Ramo-Tello 2013 [36]	3750 mg	PO	3 days	28 days	24/25
	Shaygannejad 2013 [43]	3000-5000 mg + taper	IV	13–20 days	90 days	58/64
Placebo	Rose 1970 [38]	-	IM	14 days	28 days	8/94^b^
	Durelli 1986^c^ [31]	-	IV	15 days	15 days	5.0/10^d^
	Sellebjerg 1998 [34]	-	PO	15 days	56 days	8/25

*IV* intravenous, *PO* per oral, *IM* intramuscular

^a^ Based on a weight of 70 kg

^b^ Patients were given antacids

^c^ Only the 15 days controlled period of this trial is considered here

^d^ Counts were reported per adverse event term; the number displayed here is based on an independence assumption

^e^ Patients also received 0.1 % human albumin

^f^ Includes multiple sclerosis as an adverse event

**Fig. 5:**
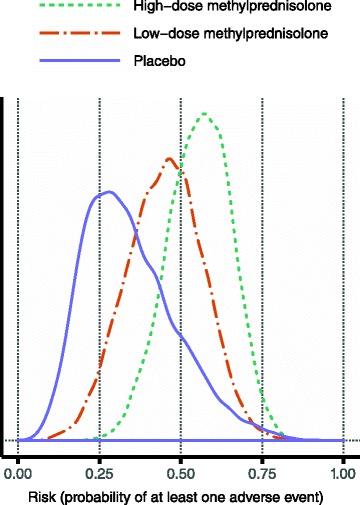
Estimated risk of non-serious adverse effects for the three alternatives. The distribution for low-dose methylprednisolone has not been estimated from data, but was obtained by random sampling of values between those sampled for placebo and high-dose methylprednisolone. The posterior median values were 0.56, 0.45 and 0.32 for high-dose methylprednisolone, low-dose methylprednisolone and placebo, respectively

The estimated distributions are displayed in Fig. 5. The distributions are again very wide, and again the expected order is seen. However, here it has been obtained by design since the risk for low-dose methylprednisolone was assumed to lie between the risks for placebo and high-dose methylprednisolone.

### Risk of serious adverse effects

The computed upper risk limits are displayed in Table 4. The aggregate numbers are high, which indicates that the limits are conservative. One possible explanation is the high threshold used for seriousness, which affects the safety margins of these limits [28]. As mentioned, for high-dose methylprednisolone only two events were reported across all investigated clinical trials. Since these studies comprise over 300 patients, even half of the computed limits are likely to be very conservative. This supports the choice in the sensitivity analysis of using uniform distributions as the most pessimistic scenario with respect to these risks.

**Table 4:** Upper risk limits computed for the various combinations of serious adverse effects and outcomes

Adverse effect	Outcome	Upper risk limit (%)	
		Low-dose methylprednisolone	High-dose methylprednisolone
Acute severe allergy	Lethal	0.24	0.00^a^
	Life-threatening	2.65	1.18
Cardio-pulmonary distress	Lethal	0.33	0.79
	Persistent	0.02	0.39
	Life-threatening	0.86	1.71
Diabetes	Lethal	0.02	0.53
	Persistent	0.86	1.05
	Life-threatening	0.07	0.13
Gastrointestinal haemorrhage	Lethal	0.26	0.39
	Persistent	0.13	0.13
	Life-threatening	0.37	0.79
Hepatotoxicity	Lethal	0.11	0.13
	Persistent	0.11	0.53
	Life-threatening	0.29	0.66
Myopathy	Persistent	0.46	0.79
	Life-threatening	0.07	0.13
Osteonecrosis^b^	Persistent	0.57	1.58
Pancreatitis	Lethal	0.04	0.13
Psychosis	Persistent	0.04	0.13
	Life-threatening	0.07	0.13
Seizure	Lethal	0.09	0.39
	Persistent	0.13	0.00^a^
	Life-threatening	0.13	0.13
Ventricular arrhythmia/cardiac arrest	Lethal	0.55	1.05
	Persistent	0.09	0.13
	Life-threatening	0.86	1.18
Total^c^	Lethal	1.65	3.42
	Persistent	2.43	4.73
	Life-threatening	5.36	6.04

^a^ This clearly is not an upper limit; therefore, in the analyses the corresponding limits for low-dose methylprednisolone are used

^b^ No requirement on the reported time to onset, due to the difficulties in diagnosing osteonecrosis

^c^ The grand total for all three outcomes is 9.44 and 14.19 % for low-dose and high-dose methylprednisolone, respectively, based on a plain summation conforming to the structure of the model, where the effects are considered to be mutually exclusive. If one instead assumes that they are independent, the total risks of experiencing any of the effects are 9.07 and 13.34 %, respectively

### Utility modelling

The utility modelling was carried out in several steps. To reduce the complexity slightly, the serious adverse effect-outcome combinations were grouped at common levels of utility. For example, all clinical outcomes consisting of a reduced relapse and a life-threatening serious adverse effect were given the same utility. This is because they are all similar in a qualitative sense, much like the non-serious adverse effects are. Among the persistent effects, osteonecrosis and diabetes were deemed least undesirable, as they are most likely not related to any life-threatening triggering event, and as they are generally manageable. At the other end of the spectrum, persistent periods of cardio-pulmonary distress, ventricular arrhythmias and seizures are likely to have been started with a life-threatening event, and should be very unpleasant and difficult to manage. Perhaps controversially, the lethal outcomes were divided into two groups, where death by pancreatitis, cardio-pulmonary distress, or gastrointestinal haemorrhage could be expected to be extended in time and very painful. In contrast, a lethal anaphylaxis or cardiac arrest should be quick with little suffering.

**Fig. 6:**
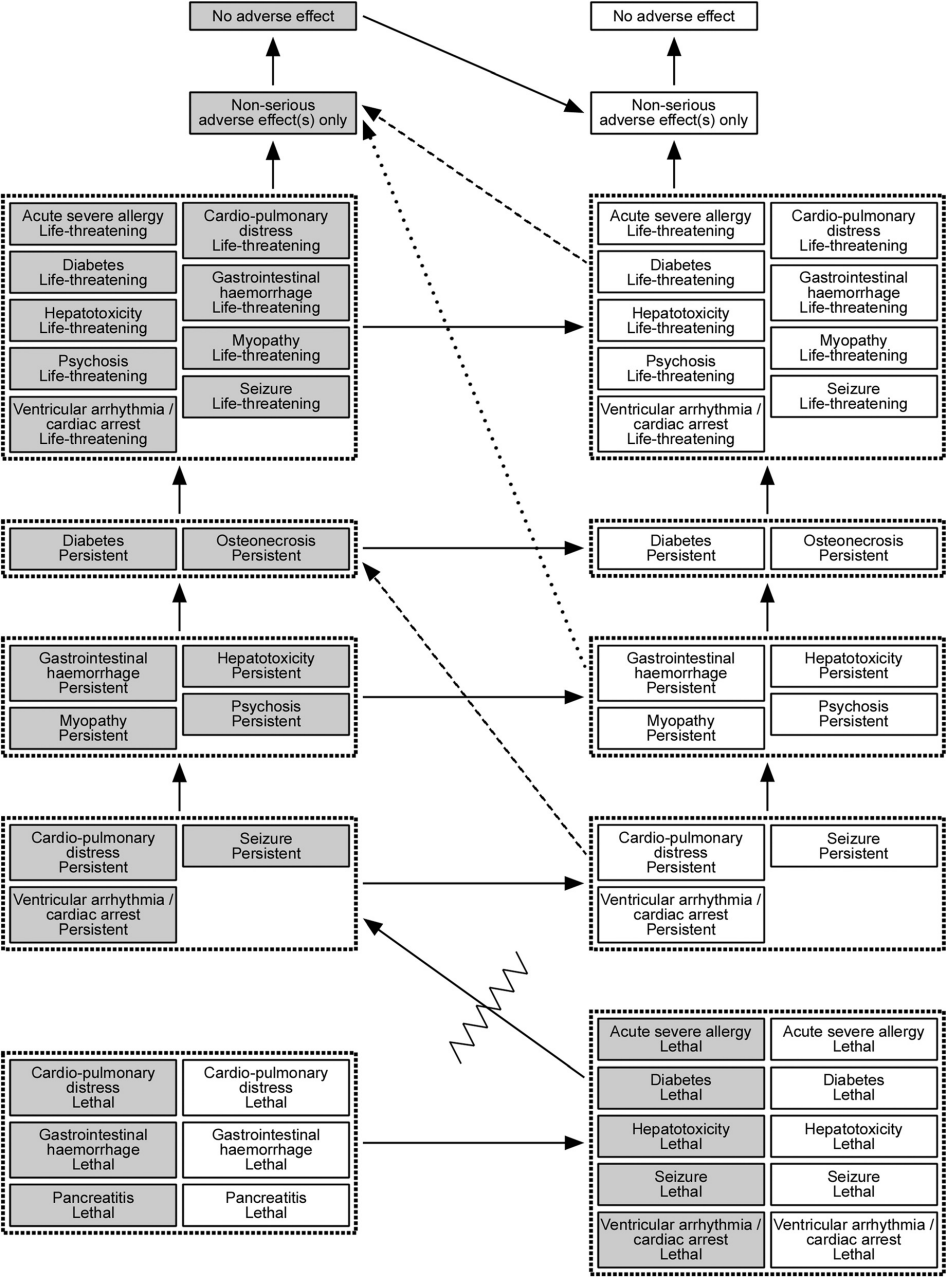
Results from the qualitative utility modelling. In the figure, grey boxes indicate clinical outcomes that include a standard relapse, i.e. no benefit, whereas white boxes signify a reduced relapse. The arrows point from a less desirable to more desirable clinical outcome. The two dashed arrows apply for patients starting their relapse at EDSS 4, and the dotted arrow for patients starting at EDSS 5. The zigzag line indicates a minimum utility difference between non-lethal and lethal outcomes

The complete results of the modelling are displayed in Fig. 6. Relations in the vertical direction are clear given the groupings just described. Relations in the horizontal direction are equally clear since the clinical outcomes are identical in terms of adverse effects but differ with respect to the beneficial effect. There are two diagonal arrows that apply regardless of the patient’s relapse severity, where one signifies the quite clear separation between non-lethal and lethal clinical outcomes, which is even assigned a minimum utility difference. The other implies that it is preferable to have a reduced relapse with a non-serious adverse event compared to having a standard relapse and no adverse event. This should be quite clear, considering what an improvement from EDSS 4 to EDSS 3 means: one has no impairments to walking compared to just being able to walk about 500 m without aid or rest. It should also be borne in mind that whereas the non-serious adverse effects are transient, the lower intensity of the MS induced by the improvement is a benefit that lasts until the end of the relapse, i.e. up to 6 months. At the same time, the difference between EDSS 4 and EDSS 3 is not immense; for example, at EDSS 4 one is still able to be ‘up and about’ for most of one’s waking hours. Therefore it should be preferable to be at that level, even with a non-serious adverse event added, compared to being at EDSS 3 and experiencing a life-threatening adverse event. Similarly, it should be preferable to be at EDSS 4 and have persistent osteonecrosis or diabetes added, compared to being at EDSS 3 and having persistent cardio-pulmonary distress or any equivalent disability added.

At EDSS 5, one is impaired to the level that one cannot work a full day, and one can walk only about 200 m without aid or rest. It was deemed reasonable that patients would prefer to remain at that level, even with a non-serious adverse event added, rather than having the reduction down to EDSS 4 and a persistent disability from the intermediate group. This group contains e.g. psychosis, which should be quite a terrifying state to endure for an extended period of time.

Clearly these latter diagonal relations are very difficult to decide upon in a general sense, and these existential choices made here should be seen primarily as rough guidance, though they were made by a very experienced physician who has encountered patients with all of these different medical problems. This framework for benefit-risk assessment could be used for the treatment of an individual patient, in which case the specific preferences of that patient should be used instead.

**Fig. 7:**
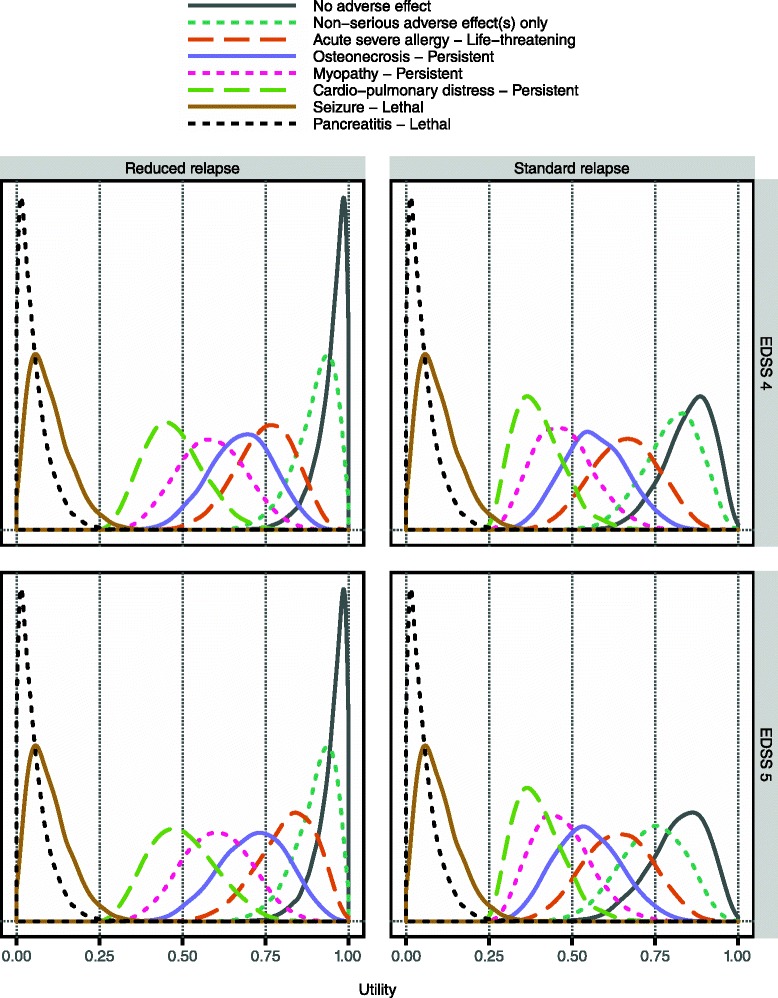
Sampled utility values for a subset of clinical outcomes. The display includes one clinical outcome from each group in Fig. 6. Clinical outcomes are divided into two panel columns according to whether or not they entail the considered beneficial effect. Also, results are shown separately by panel row according to relapse severity as measured by initial EDSS. (Cf. the different relations indicated in Fig. 6 for patients starting their relapses at EDSS 4 and 5, respectively.) Here, the minimum utility difference between non-lethal and lethal outcomes has been set to 0.25

The resulting distributions guided by these qualitative relations are shown in Fig. 7. They appear to convey reasonably well the intents of the utility modelling. It should be noted that lack of benefit (i.e. ‘standard relapse’) in combination with no or only non-serious adverse effects has a notably lower utility if the relapse starts at EDSS 5 than if it starts at EDSS 4. This is clinically sensible, and should imply that treatment effectiveness is more rewarded for more severe relapses.

### Evaluation results

**Fig. 8:**
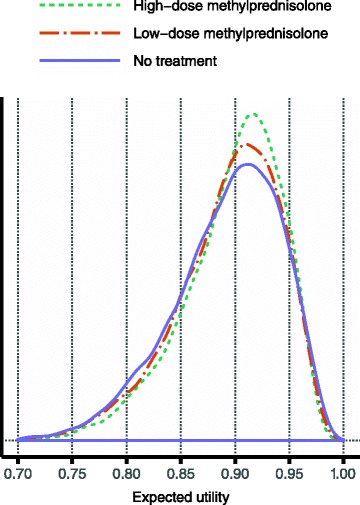
Resulting expected utility distributions in one specific sensitivity analysis scenario. These distributions of expected utilities were obtained for the three considered alternatives in the following scenario: over the derived risk intervals for serious adverse effects the truncated *Exponential (5/upper limit)* distribution was applied; the proportion of risk attributed to the background for serious adverse effects was set to 10 %; the minimum utility difference between non-lethal and lethal outcomes was 0.4; and the utility modelling was for patients starting their relapse at EDSS 4. The median values of the resulting expected utilities were 0.91, 0.90 and 0.90 for high-dose methylprednisolone, low-dose methylprednisolone and the no treatment alternative, respectively

**Fig. 9:**
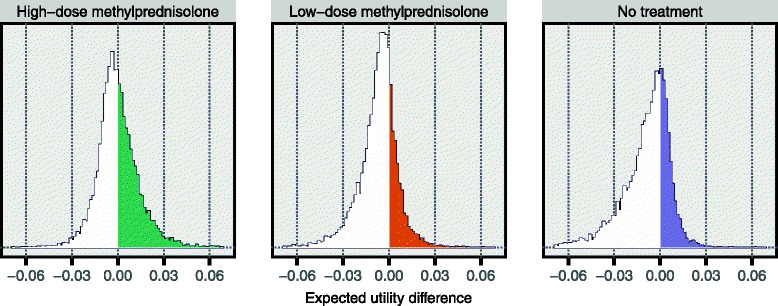
Resulting preference rates in one specific sensitivity analysis scenario. These histograms are based on the same results as those presented in Fig. 8. In each panel, the difference in expected utility was computed between the alternative indicated above the panel and the maximum expected utility for the other two alternatives. Using the left panel as an example, in each of the 10,000 iterations a difference was computed between the sampled expected utility for high-dose methylprednisolone and the highest expected utility of those sampled for low-dose methylprednisolone and the no treatment alternative. The 10,000 values for the difference thus obtained were then used to construct the displayed histogram. This means that the proportion of this histogram that is to the right of zero, i.e. the coloured proportion, is the fraction of all iterations in which high-dose methylprednisolone had the highest expected utility. Hence, this is precisely the preference rate for high-dose methylprednisolone, in this specific sensitivity analysis scenario. (See Fig. 1 for a definition of the preference rate.) Here, the preference rates are 45, 25 and 30 % for high-dose methylprednisolone, low-dose methylprednisolone and the no treatment alternative, respectively

As indicated in Fig. 1, once the structure of the model has been specified, and once distributions are available for all constituent probability and utility variables, it is possible to compute expected utilities for the considered alternatives over the iterations of the probabilistic analysis. Figure 8 shows how the resulting expected utility of the respective alternatives was distributed in one specific sensitivity analysis scenario. While the graphs superficially suggest very small differences between the alternatives, Fig. 8 fails to recognise the many inter-dependencies that exist between variables of this assessment. These dependencies imply that proper inference requires comparisons to be made at the iteration level prior to aggregating the results. Specifically, Fig. 9 is based on the differences in expected utility obtained over the 10,000 iterations. This figure illustrates the concept of the preference rate and shows much clearer than Fig. 8 the comparative results for the alternatives.

**Fig. 10:**
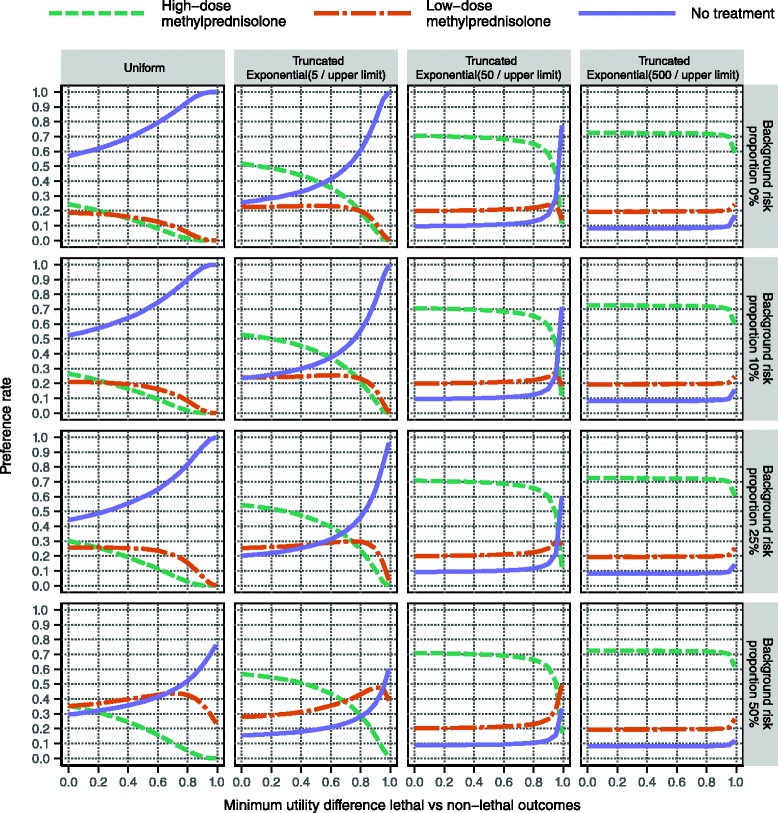
Evaluation results based on the utility modelling for patients starting at EDSS 4. Within each panel, the alternatives’ preference rates are shown at varying levels of the minimum utility difference. Distributions over the risk intervals for serious adverse effects are varied column-wise, with risks generally decreasing to the right (see main text). The proportion of risk attributed to the background for serious adverse effects is increased by row downwards. As an example, the preference rates presented in Fig. 9 are found in the second row and the second column, at the minimum utility difference 0.4

The main finding in the evaluation results across all considered sensitivity analysis scenarios is the inferiority of low-dose methylprednisolone: it was the preferred alternative in less than 5 % of all scenarios, and in no single scenario was its preference rate above 50 %. This finding is visually evident in Fig. 10, which displays the results based on the utility modelling for less severe relapses starting at EDSS 4. Essentially, high-dose methylprednisolone and the no treatment alternative alternate as the option with the highest preference rate, depending on the setting of the sensitivity analysis variables. As the distributions over the risk intervals for serious adverse effects become more and more skewed towards lower risks (cf. Fig. 2), the more preferable high-dose methylprednisolone becomes: this is evident by comparing the panel rows from left to right. In contrast, as the minimum utility difference between non-lethal and lethal outcomes is increased, the preference rate of high-dose methylprednisolone decreases substantially: this effect is visible in every panel of the display. The reason is that as this minimum difference increases, so does the penalty incurred by the active treatment alternatives for their higher risk of lethal outcomes. The least impacting of the considered sensitivity analysis variables was the proportion of risk attributed to the background for serious adverse effects: results change only modestly over the various panel rows.

The minimum utility difference is in itself quite opaque. However, some aid to its interpretation is possible by specifying three clinical outcomes and translating the utility difference into a gamble including those outcomes [15]. Specifically, from the sampled utility values one can determine at what point the typical patient becomes indifferent between the status quo outcome (a standard relapse without adverse effects) and gambling between the best possible outcome (a reduced relapse without adverse effects) and the worst possible outcome (lethal pancreatitis or any of its equivalents in Fig. 6). For example, in Fig. 10 a minimum utility difference of 0.5 corresponds to indifference between status quo and gambling with a probability for the lethal outcome of about 7 %, and therefore a probability of about 93 % for the best possible outcome. At a minimum utility difference of 0.9, the typical patient is more risk-averse and requires the probability of the lethal outcome to go down to about 1 % before considering the gamble equivalent to the status quo outcome.

**Fig. 11:**
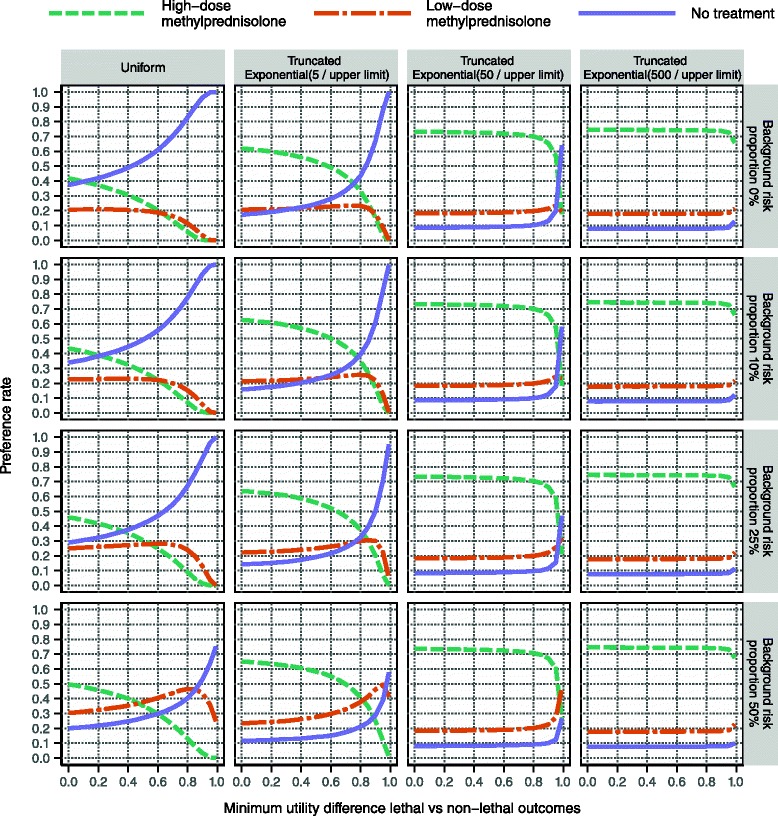
Evaluation results based on the utility modelling for patients starting at EDSS 5. This display is analogous to that in Fig. 10, albeit based on a scenario that considers more severe relapses

Figure 11 displays the same types of results as Fig. 10, though based on the utility modelling for more severe relapses starting at EDSS 5. Whereas the overall conclusions are the same, the results show that when everything else is kept constant, high-dose methylprednisolone is more likely to be the preferred alternative when the relapse is severe. This makes sense clinically and fits with the observations from the utility distributions in Fig. 7. (See Section ‘Utility modelling’).

**Fig. 12:**
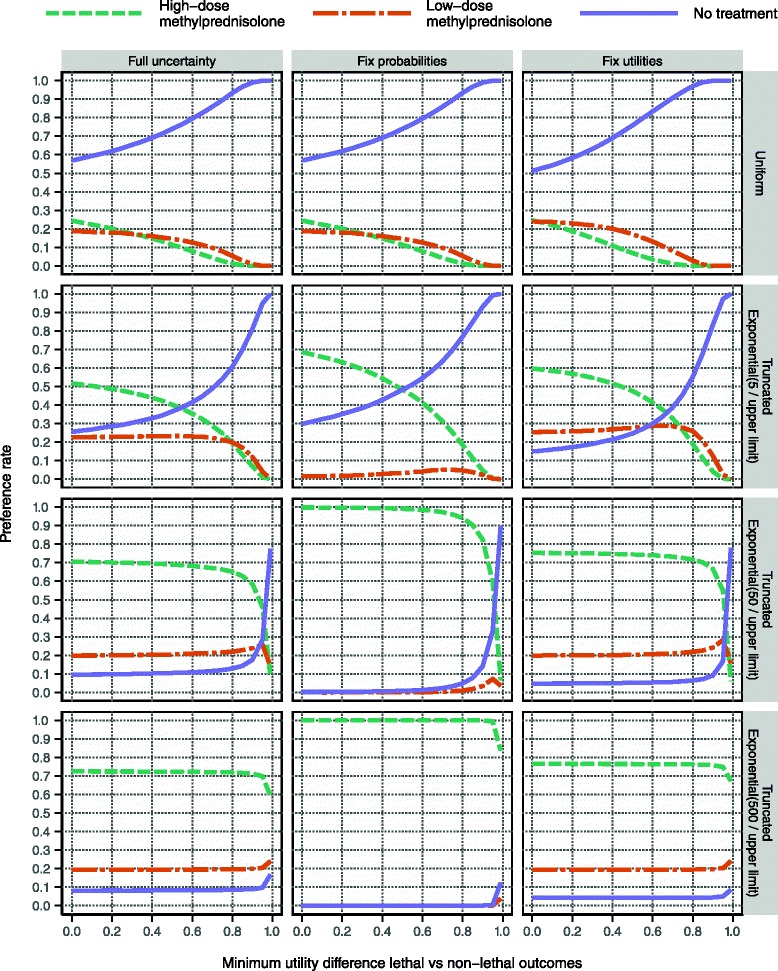
Evaluation with respect to various sources’ contribution to overall uncertainty. This display, like the one in Fig. 10, is based on the utility modelling for patients starting at EDSS 4. The background risk proportion for serious adverse effects is kept constant at zero, which means that the left column here is identical to the top row in Fig. 10. This reference is compared to two sets of analyses: the removal of all sampling uncertainty from probability variables and utility variables, respectively, in the middle and right columns. This fixation effect was achieved by replacing all sampled values of a particular variable by the median of the sampled values for that variable

One observation is that high-dose methylprednisolone never reaches a preference rate above 75 % in any of the investigated scenarios, which suggests that some aspect of the assessment contains too much uncertainty to clearly recommend high-dose methylprednisolone. In Fig. 12 it is demonstrated that the removal of sampling uncertainty from all utility variables has only a modest effect on the maximum preference rate. However, the removal of uncertainty from probability variables has a much more profound effect. In particular, high-dose methylprednisolone does reach a 100 % preference rate when the risks for serious adverse effects are kept at a fixed minimal level, as seen in the bottom panel row in Fig. 12. This result is coherent with the wide distributions for probability variables presented in Figs. 4 and 5.

## Discussion

This is the first ever assessment of methylprednisolone in MS relapses that considers both the effectiveness of treatment and its risk for adverse effects. Our results favour a high-dose (at least 2000 mg) short-term regimen of methylprednisolone over one with doses below 1000 mg. Although there is a paucity of data, especially for the low-dose alternative, our results are reassuring with respect to current treatment recommendations and clinical practice.

The subsequent discussion addresses, in turn, study design choices, methodological issues and related work.

### Study design choices

This assessment considers a single active treatment, given at two different doses. Corticotropin was not included, as it has been essentially abandoned due to its impractical administration. Dexamethasone has been studied only to a limited extent [26, 27], and there was too little data for it to be considered here. The same applies for plasma exchange, which has been proposed as possible second-line treatment [1]. Intravenous immunoglobulin does not appear to be effective in MS relapses [1].

Our definitions of high- and low-dose methylprednisolone are by necessity arbitrary, given that no generally accepted definitions exist. While the dose that strikes the optimum balance between benefit and risk may not conform to either of these definitions, they do have the advantage of offering two clearly separated alternatives, which facilitates assessment. Also, it appears that few studies to date have been concerned with doses in the intermediate dose range excluded from consideration here. Finally we note that existing treatment recommendations agree with our adopted definition of high-dose methylprednisolone [1], and that the resulting dose classifications in previous analyses conform with ours [2].

In previous appraisals of glucocorticoids for MS relapse management, there has been a strong emphasis on route of administration [9, 10]. Pharmacologically, the bioavailable dose should be far more important than the route of administration, for which reason it is surprising that the oral bioavailability of methylprednisolone has not been ascertained. Only one study compared the bioavailability of oral prednisone and intravenous methylprednisolone at equivalent doses, and could not demonstrate any difference after 48 h [44]. The lowest per-oral dose from any study considered as high-dose methylprednisolone in this assessment was 3676 mg. Hence, this dose would qualify as high according to our definition so long as the bioavailability could be assumed to be 55 % or higher. Although the quoted study [44] included only 16 patients and used a chemically similar but not identical glucocorticoid, this assumption seems very reasonable. Nevertheless, our assessment framework is transparent and flexible enough that a re-assessment based on route of administration rather than dose would be easily possible.

In this analysis, estimation of effectiveness relies on EDSS values assessed sometime between 14 and 28 days from start of treatment. Our target time point of 28 days could possibly be too early to capture the full extent of the treatment effects. However, this design choice is advantageous for the power of the analysis, since it allows inclusion of studies that lack long-term follow-up. Also, across different studies, the consistency in the actual assessment time points would likely decrease with a later target time point. On the whole, 28 days appears to be a reasonable choice, although it too could be altered within the employed assessment framework.

#### Exclusion of optic neuritis

The biological link between MS and optic neuritis is unquestionable [45], even though clinically isolated optic neuritis is neither a sufficient nor a necessary condition for the diagnosis of MS [46]. Solid arguments can be made for any of the following plausible alternative designs: analysing MS relapse patients only, analysing MS relapse and acute optic neuritis patients in parallel, or analysing both groups jointly. As with the other design choices discussed above, our framework could accommodate either alternative, if the appropriate data is provided.

The alternative comprising parallel, but separate, assessments with the same overall methodology would be an appealing complementary study: an interesting idea for further research.

As regards the alternative of conducting a joint assessment, such a design would have benefitted the power of our analysis. However, it also would have created two rather severe analytical obstacles. First, major clinical trials of glucocorticoids in acute optic neuritis include only a very limited number of patients diagnosed with MS [47, 48], thus introducing an important source of heterogeneity and potential bias. A clear majority of patients, even among those on placebo, improve their EDSS quickly [48], which supports the notion that these patients are in an earlier phase of their clinical course and therefore qualitatively different compared to the patients included in MS relapse trials.

Secondly, as far as we are aware there is only a single acute optic neuritis trial that reports outcomes in terms of EDSS improvement [48]. The others report only visual outcomes, which are non-trivial to translate into equivalents of EDSS improvement, both conceptually but also practically as the translation would require patient-level data.

### Methodological issues

The literature search strategy in this assessment is slightly unorthodox: it considers studies that have been included in earlier systematic reviews of glucocorticoids in MS, or that have referred to any such review. Our coverage up to November 2009 is at least as good as that of a dedicated European Federation of Neurological Societies task force, which scanned the literature at that point in time [22]. Studies published later than that would be missed if they did not refer to any of the seven reviews considered here [1, 2, 8–10, 21, 22], and were not investigated in the 2012 Cochrane review by Burton et al. [9]. This risk should be small.

When estimating effectiveness and risk of non-serious adverse effects, any study was included that contained at least one arm corresponding to any of the considered treatment alternatives. This deviates from the usual meta-analysis strategy, where only such studies are included that contain all treatments of interest. A pragmatic motivation for our approach is that no single study was identified that compared both low- and high-dose methylprednisolone to placebo. Likewise, a head-to-head comparison of only high- and low-dose methylprednisolone was not feasible since the risk for non-serious adverse effects was not directly estimable from data for the low-dose alternative. Apart from these pragmatic considerations, it should be noted that since decision analysis uses per-alternative probabilities, there is no intrinsic requirement on included studies to contain all treatment alternatives. On the contrary it can be argued that omitting a study that misses one or more alternatives would be a waste of information concerning the alternatives that are in fact included in that study: after all, studies were required to fulfil certain pre-defined inclusion criteria, which should provide a baseline level of homogeneity across all studies.

The overall framework in this benefit-risk assessment is decision analysis, which has been recommended elsewhere [12]. The use of probabilistic evaluation is mandated by one of the leading bodies for health technology assessment globally [49]. Within this framework, two novel methods are used in this assessment: one for utility modelling [15], and one for deriving limits on the risk of serious adverse effects [28]. The former method has certain advantages: it is relatively quick; it requires neither utility elicitation studies nor collection of external utility estimates; and it avoids many of the assumptions inherent to time-aggregating utility metrics like the quality-adjusted life year [15]. At the same time, qualitative relations can only carry so much information, and typically minimum utility differences must be used. This requires additional sensitivity analysis scenarios, which may make interpretation more difficult.

The use of risk limits computed from individual case reports was required since no suitable risk estimates were publicly available. These limits are valid only under certain assumptions, which are likely to be fulfilled here (see Additional file 4). Because the overall evaluation results were highly sensitive to the distribution of risks up to their respective limits, it would be worthwhile to investigate whether more precise risk estimates could be obtained from another source. One candidate would be a large, possibly multi-national, repository of longitudinal patient records.

### Limitations

This assessment has several limitations, of which most can be attributed to a paucity of data in general, and a complete absence of appropriate data in specific aspects.

Clinical trials measure efficacy, which seldom corresponds precisely to effectiveness seen in real-world clinical use. Hence, in this assessment effectiveness is likely to be overestimated for all alternatives, and unpredictably so. Clinical trial patients on placebo are probably more unlike patients in clinical practice who abstain from treatment than are clinical trial patients on active treatment in relation to their clinical practice counterparts. Publication bias, if present, will most likely selectively overestimate the effectiveness of active treatment.

The risk of non-serious adverse effects from low-dose methylprednisolone could not be estimated from data. Instead, risk values were sampled uniformly from the intervals formed by the values sampled for the other two alternatives. The resulting average placement of low-dose methylprednisolone at equal distance from placebo and high-dose methylprednisolone is likely to underestimate its true risk: the doses classified as low here are not low in an absolute sense, and are certainly high enough to induce non-serious adverse effects typical for glucocorticoids, e.g. insomnia and oedema.

The available data did not permit probability variables, in particular effectiveness, to be estimated separately for patients with differentially severe relapses. This would have been highly desirable given that such differentiation was used in the utility modelling. Further, potentially important covariates such as age, gender, concomitant medication and pre-relapse disability could not be taken into account since patient-level data from the included studies were not provided, even after request.

Benefit was in this assessment defined as an improvement of at least one point on the EDSS. While this definition has the advantage of being commonly used and thus avoids unnecessary exclusion of potential studies, it also has certain limitations. First, it is contingent on the EDSS itself. This implies that only clinical assessment of patient disability is considered, while other aspects reflected by e.g. quantitative tests of neurological performance or patient-reported outcomes are disregarded [50]. Secondly, there is no differentiation with respect to the degree of recovery. This would have required patient-level data; however, even if such data had been accessible, the ordinal nature of the EDSS would have severely complicated the analysis of variable degrees of recovery.

A limitation with all included analyses based on the individual case reports in VigiBase is that they represent patients with mixed indications. A restriction to MS patients only was not feasible since the indication was very often not stated in the reports, which would have caused too severe a loss of data on harms.

### Related work

Although there is no prior benefit-risk assessment of methylprednisolone in MS relapses, the meta-analysis by Miller et al. [2] is highly relevant in relation to our results for the respective alternatives’ effectiveness. Miller et al. conclude that high-dose methylprednisolone is more effective than placebo but equally effective as low-dose methylprednisolone. However, in this assessment the high-dose regimen is clearly more effective than its low-dose comparator, as seen in Fig. 4.

Two possible explanations have been identified that could explain this discrepancy. Miller et al. use as their endpoint the mean change in EDSS rather than the fraction of improved patients; and they include only two studies, namely those where high- and low-dose methylprednisolone are compared head to head (cf. the discussion under ‘Methodological issues’). The latter discrepancy is likely to be the most important, given the results obtained by Barnes et al. [33], which contributed 80 % of all patients in the analysis by Miller et al. Using our definition, high-dose methylprednisolone had an effectiveness of 0.34 in the study by Barnes et al., which is a value that deviates considerably from the seven other high-dose arms considered: none of those had a value below 0.5, and the posterior median value from all eight study arms combined was 0.64. At the same time, the value for low-dose methylprednisolone in that same study was 0.48, which is very close to the overall estimated effectiveness for that alternative. Hence, it seems that Miller et al. have grossly underestimated the effectiveness of high-dose methylprednisolone by including only studies where it was compared head to head with low-dose methylprednisolone, which led to selecting a highly unrepresentative study as the main contributor to their pooled results. Our results in Fig. 4 correspond to a dose–response relationship that is pharmacologically plausible. Also, it seems that our results comply with experience from clinical practice: if the low-dose regimen had been perceived as equally effective, it would probably have been used more often, as it could be expected to be favourable on the risk side.

## Conclusions

Over the numerous sensitivity analysis scenarios considered in this quantitative benefit-risk assessment of methylprednisolone in MS relapses, the low-dose regimen of less than 1000 mg over at most 31 days was rarely the preferred alternative. And when it was, the level of confidence in its status as most preferred was not great. Hence, based on the available information, a change of treatment recommendation from high- to low-dose methylprednisolone in this indication cannot be justified. However, it must be borne in mind that the risk of non-serious adverse effects was not evaluable from data for low-dose methylprednisolone, and its effectiveness was estimated based on only three trials comprising merely 62 patients in total.

Overall, our results were not able to differentiate between the high-dose methylprednisolone regimen of at least 2000 mg over at most 31 days and the no treatment alternative. The more skewed towards zero the risk distributions for serious adverse effects, and the less risk-averse the patient population, the more favourable were the results for high-dose methylprednisolone. However, the considerable posterior uncertainty in the estimates of effectiveness and risk of non-serious adverse effects denied high-dose methylprednisolone a higher preference rate than 75 % in any sensitivity analysis scenario. All of this, in addition to the severe paucity of data for low-dose methylprednisolone discussed above, suggests that more clinical research is needed. Any clinical neurologists should feel compelled to assist in this process to optimise the treatment of MS using corticosteroids, e.g. by contributing patients to clinical trials, submitting well-documented case reports of suspected adverse reactions, or carefully managing patients’ health records to make them as useful as possible for research purposes.

Our results clearly indicate that methylprednisolone treatment is more likely to be the right decision in severe MS relapses, which makes sense from a clinical point of view. This finding also highlights sensitivity in the overall results to the particular relations used in the utility modelling. An important implication is that for an individual patient, this assessment can serve merely as a starting point to guide treatment, and his or her specific preferences should be carefully considered in the decision.

The overall superiority of high-dose methylprednisolone relative to its low-dose comparator was seen in spite of additional risks with higher doses, e.g. for hepatotoxicity [6]. However, we wish to emphasise the importance of considering such small but possibly significant risks in the management of individual patients: if the adverse effect does set in, it must be recognised and managed, and alternative treatments must be considered.

## Availability of supporting data

The data sets supporting the results of this article are available from the Dryad Digital Repository, [http://dx.doi.org/10.5061/dryad.fh2nt].

## Additional files

Additional file 1:
**Methylprednisolone dose calculations for individual case reports in VigiBase.** (PDF 185 kb) Additional file 2:
**Definitions of serious outcomes on individual case reports in VigiBase.** (PDF 143 kb) Additional file 3:
**Markov Chain Monte Carlo simulation of probability variables corresponding to effectiveness and risk of non-serious adverse effects.** (PDF 248 kb) Additional file 4:
**Computation of upper risk limits for serious adverse effects based on individual case reports in VigiBase.** (PDF 193 kb) Additional file 5:
**Sampling from qualitatively modelled utility variables in probabilistic decision analysis.** (PDF 155 kb) Additional file 6:
**Definitions of included serious adverse effects.** (PDF 111 kb) Additional file 7:
**Process for selecting published studies used to estimate effectiveness and risk of non-serious adverse effects.** (PDF 97 kb)
