# Supplementary material for: Quantitative benefit-risk assessment of methylprednisolone in multiple sclerosis relapses
Source: BMC Neurol. 2015 Oct 16;15:206. doi: 10.1186/s12883-015-0450-x (PMC4609048; doi:10.1186/s12883-015-0450-x)
Supplement: Additional file 1: — Methylprednisolone dose calculations for individual case reports in VigiBase. (PDF 185 kb) [file 12883_2015_450_MOESM1_ESM.pdf]

**Additional file 1: Methylprednisolone dose calculations for individual case reports in VigiBase®**

*Materials and methods*

This study employed a VigiBase extract from May 2012, from which suspected duplicates were removed [1]. Any remaining report was considered that contained at least one drug either coded directly as methylprednisolone, or coded with any medicinal product mapped to methylprednisolone at the generic level of the WHO Drug Dictionary Enhanced™ [2]. For each report, it was assessed whether the total duration of methylprednisolone use was 31 days or less, and, if so, whether the accumulated methylprednisolone dose was either below 1,000 mg or at least 2,000 mg. This corresponds to the definitions for low- and high-dose methylprednisolone, respectively; for details, please see the main article. The assessment process was primarily automatic as described below.

For reports with more than one listing of methylprednisolone, all entries were compared pairwise to detect and remove duplicates. On reports with more than one unique methylprednisolone entry, it was required first that all start and stop dates be provided, and secondly that the time difference between the earliest of all start dates and the latest of all stop dates be at most 31 days. On reports with only a single methylprednisolone entry, the actual start and stop dates were not required, so long as the duration as such was still assessable. For example, the duration may have been coded without any dates, or it may be calculable as the ratio between total and daily dose.

The accumulated dose was assessed for each methylprednisolone entry separately, and then summed up to get a total per each report. For a given methylprednisolone entry, three different approaches were employed sequentially to extract the dose automatically. First, an attempt was made to calculate the accumulated dose based entirely on the coded structured data. This calculation considered one or more of the following data fields: duration, frequency of administration (with unit), dose (with unit) at each administration, total dose, patient weight, and patient height. If that failed, minor assumptions were made regarding missing data that could potentially make the dose calculable. For example, information is often omitted if considered to correspond to the default value, which for frequency is one. Hence ‘per day’ was assumed to mean ‘once per day’ unless explicitly stated otherwise. If the dose was still not calculable after the introduction of such minor assumptions, an attempt was made to extract it from the free text information corresponding to that particular methylprednisolone entry, if available. If no dose could be extracted, the automatic dose assessment was considered unsuccessful. As a last attempt, if there was any free text information available for the report as a whole, that text was manually reviewed in an attempt to assess the total methylprednisolone dose.

The entire dose assessment process was tested for its accuracy by randomly selecting 50 reports for manual review. The selection was stratified in such a way that doses extracted from free text were overrepresented, as they were deemed at higher risk of being incorrectly assessed.

*Results*

There were 29,750 methylprednisolone reports in total, of which methylprednisolone was characterised as a suspected (S) or interacting (I) drug on 12,170 reports. 778 reports (554 for SI) were classified as high-dose methylprednisolone, while 4,548 reports (2,593 for SI) were classified as low-dose methylprednisolone. The distribution of reports across the different methods of dose assessment is displayed in Table A1.

With respect to the classification into dose groups, the automatic dose assessment agreed with the manual review for all 50 randomly selected reports.

**Table A1. Number of VigiBase reports on high- and low-dose methylprednisolone.**

| Drug role characterisation[a] | Dose group | Method of dose assessment | Number of reports |
|---|---|---|---|
| Suspected or interacting | High | Structured data without assumptions | 333 |
| Suspected or interacting | High | Structured data with assumptions | 121 |
| Suspected or interacting | High | Automatic free text extraction | 84 |
| Suspected or interacting | High | Manual free text extraction | 16 |
| Suspected or interacting | Low | Structured data without assumptions | 1,351 |
| Suspected or interacting | Low | Structured data with assumptions | 906 |
| Suspected or interacting | Low | Automatic free text extraction | 335 |
| Suspected or interacting | Low | Manual free text extraction | 1 |
| Concomitant | High | Structured data without assumptions | 148 |
| Concomitant | High | Structured data with assumptions | 60 |
| Concomitant | High | Automatic free text extraction | 16 |
| Concomitant | Low | Structured data without assumptions | 730 |
| Concomitant | Low | Structured data with assumptions | 1,191 |
| Concomitant | Low | Automatic free text extraction | 34 |

[a] A report was categorised as 'Suspected or interacting' if at least one of its methylprednisolone entries was characterised as such.

*References*[*]


1. Norén GN, Orre R, Bate A, Edwards IR. Duplicate detection in adverse drug reaction surveillance. Data Min Knowl Discov. 2007;14:305-28.
2. Lindquist M. VigiBase, the WHO Global ICSR Database System: Basic facts. Drug Inf J. 2008;42:409-19.


[*] Reference number 2 corresponds to reference number 20 of the main article to which this additional file serves as supporting information.
