# Supplementary material for: Quantitative benefit-risk assessment of methylprednisolone in multiple sclerosis relapses
Source: BMC Neurol. 2015 Oct 16;15:206. doi: 10.1186/s12883-015-0450-x (PMC4609048; doi:10.1186/s12883-015-0450-x)
Supplement: Additional file 2: — Definitions of serious outcomes on individual case reports in VigiBase. (PDF 143 kb) [file 12883_2015_450_MOESM2_ESM.pdf]

Additional file 2: Definitions of serious outcomes on individual case reports in VigiBase®

In this study, a set of different serious adverse effects were considered. As detailed in Additional file 6, each effect was defined from a set of MedDRA preferred terms. Most often an effect was considered to be present on a report if any of its corresponding terms was listed, although sometimes two terms were required to be co-listed.

Within the context of this benefit-risk assessment, a serious adverse effect was defined as an effect resulting in life-threatening and/or persistently disabling reactions, where death is an obvious possible outcome of a life-threatening reaction. The included terms and term combinations were together intended to capture all reported events suspected to have resulted from the various considered serious adverse effects. However, not all occurrences of those adverse effects were necessarily life-threatening or caused persistent disability. There was therefore a need to strictly define what is meant by lethal, persistent, and life-threatening (non-lethal) outcomes, respectively, in terms of information available on the individual case reports used in this study. In particular, those were the three serious outcomes considered in the decision model constructed for the benefit-risk assessment at hand.

Lethal

- Basically, a lethal outcome was inferred if any available information on the report suggested the patient died. The following criteria were considered:
    - Co-reported preferred terms: ‘Death neonatal’, ‘Death’, ‘Sudden infant death syndrome’, ‘Sudden death’, or ‘Completed suicide’
    - ‘Death’ was coded as the reason for seriousness
    - The cause of death was reported
    - The date of death was reported
    - An autopsy result was reported
    - The reported outcome of any listed term was ‘Died’, ‘Died - reaction may be contributory’, or ‘Died - unrelated to reaction’
- The only exception to the above rule was explicit information for the term(s) specific to the adverse effect under consideration suggesting the outcome of that particular effect was in fact not lethal for this patient:
    - Reported outcome was ‘Died - unrelated to reaction, ‘Recovered’, ‘Recovering’, ‘Not recovered’, or ‘Recovered with sequelae’

Persistent

- A persistently disabling outcome was inferred on a report if any of the following five criteria was fulfilled:
    - At least one of the reported terms specific to the adverse effect under consideration was defined as intrinsically persistent in Additional file 6
    - ‘Disabling’ was coded as the reason for seriousness, except if the outcome for the adverse effect-specific term(s) was explicitly reported as ‘Recovered’

    - The outcome of any of the adverse effect-specific terms was reported as ‘Recovered with sequelae’
    - The duration of at least one of the adverse effect-specific terms was coded with complete dates and was at least 90 days
    - The outcome of at least one of the adverse effect-specific terms was coded as ‘Not recovered’ or ‘Recovering’ and its reaction onset date was complete and preceded the reporting date by at least 90 days

Life-threatening

- A life-threatening (non-lethal) outcome was inferred on a report if any of the following two criteria was fulfilled:
    - At least one of the reported terms specific to the adverse effect under consideration was defined as intrinsically life-threatening in Additional file 6
    - The reason for seriousness was coded as ‘Life-threatening’ or ‘Other’

It follows that any of the considered serious adverse effects could be associated with more than one serious outcome on the same report. (For example, ‘Death’ could be co-reported with a term intrinsically assigned as life-threatening.) To overcome this issue, a hierarchical classification was applied in the order provided above. In other words, an adverse effect under consideration could not be classified as persistent or life-threatening on a report where it already fulfilled the requirements for the lethal outcome. Similarly, it could not be classified as life-threatening if already meeting the requirements for persistent.

Clearly, more than one serious adverse effect can be present on the same report. Depending on the other information provided on the report, two different adverse effects could be associated with different serious outcomes. (Obviously, it could also happen that only one of the two effects was associated with any of the three outcomes in the first place.)
