# Supplementary material for: Quantitative benefit-risk assessment of methylprednisolone in multiple sclerosis relapses
Source: BMC Neurol. 2015 Oct 16;15:206. doi: 10.1186/s12883-015-0450-x (PMC4609048; doi:10.1186/s12883-015-0450-x)
Supplement: Additional file 3: — Markov Chain Monte Carlo simulation of probability variables corresponding to effectiveness and risk of non-serious adverse effects. (PDF 248 kb) [file 12883_2015_450_MOESM3_ESM.pdf]

**Additional file 3: Markov Chain Monte Carlo simulation of probability variables corresponding to effectiveness and risk of non-serious adverse effects**

*The hierarchical beta-binomial model*

In this assessment, effectiveness was estimated by the fraction of patients experiencing a clinical improvement of at least one point on the expanded disability status scale (EDSS). Similarly, risk of non-serious adverse effects was estimated by the fraction of patients experiencing at least one non-serious adverse effect. For the various considered treatment alternatives, respectively, both of these probability variables were estimated based on a series of treatment arms from studies included on pre-specified criteria.

The hierarchical beta-binomial model is a natural and commonly used model to combine results from a set of parallel experiments concerning a binary outcome [1]. Hence it is suitable to analyse the data available for effectiveness and risk of non-serious adverse effects in this assessment. Because it is a model from Bayesian statistics, inference is based on a posterior distribution. This fits well with the probabilistic decision-analytical framework employed in the current benefit-risk assessment.

Consider the *i*:th treatment alternative and its $j = 1,2,\ldots,J$ available study arms, each with $n_j^i$ included patients among which $y_j^i$ experience clinical improvement as specified above. The model presupposes binomial distributions for each of the study arms separately:

$$y_j^i \mid p_j^i \sim Bin(n_j^i, p_j^i) \qquad (1)$$

where $p_j^i$ is the probability for a patient in the *j*:th study arm for the *i*:th treatment alternative to experience clinical improvement. Contrary to standard meta-analysis, there is no strict homogeneity assumption; for example, patient populations are not assumed to be identical in nature across the study arms. Rather, the study arms are linked through the assumption of a common higher-level distribution for the probability to experience clinical improvement:

$$p_j^i \mid \alpha^i, \beta^i \sim Beta(\alpha^i, \beta^i) \qquad (2)$$

where the hyperparameters $\alpha^i$ and $\beta^i$ determine both the central tendency of the probabilities $p_j^i$ for the different study arms, as well as the variability between them. As the overall measure of the effectiveness of the *i*:th treatment alternative we use the expectancy of these probabilities:

$$p^i = E\left[p_j^i\right] = \frac{\alpha^i}{\alpha^i + \beta^i} \qquad (3)$$

It should be noted that both $\alpha^i$ and $\beta^i$, and therefore $p^i$, are random parameters. Probabilistic inference is possible in the Bayesian setting via the posterior distribution of $p^i$, as detailed below. However, this requires not only data from the various included study arms, but also a prior distribution for $\alpha^i$ and $\beta^i$. Here, a non-informative joint prior distribution is employed:

$$f\left(\alpha^i, \beta^i\right) \propto \frac{1}{\left(\alpha^i + \beta^i\right)^{5/2}} \qquad (4)$$

The model specification in Equations 1 – 4 applies to the effectiveness of the various considered treatment alternatives. The exact same mathematical framework was used for the risk of non-serious adverse effects, though with the variable $y_j^i$ in Equation 1 instead indicating the number of patients experiencing at least one non-serious adverse effect. Also, from a practical perspective, study arms were included based on different criteria, as described in the main article.

*Sampling from the posterior distribution of $p^i$*

For the purpose of sampling posterior values for the various probabilities $p^i$ to be used in the probabilistic benefit-risk assessment, Markov Chain Monte Carlo (MCMC) simulation was employed. Specifically, the Metropolis-Hastings algorithm [2, 3] was used, since it is well suited to handle the complex posterior distributions of the hierarchical beta-binomial model. Each $p_j^i$ was reparameterised as the logarithm of the odds $p_j^i/(1-p_j^i)$, and each pair of $\alpha^i$ and $\beta^i$ was reparameterised as the pair $(\log \alpha^i - \log \beta^i)$ and $\log(\alpha^i + \beta^i)$. All jumping distributions were normally distributed.

The practical simulation strategy followed closely that of expert recommendations [1]. The variances of the jumping distributions were tuned to obtain near-optimal rates of accepted jumps. Twenty parallel MCMC chains were used with starting points randomly dispersed around crude estimates of $\alpha^i$ and $\beta^i$. For each chain, the first 5,000 samples were discarded; thereafter every tenth sample was retained until 500 samples had been generated in total. Thus, 10,000 values were sampled in total for each $p^i$. Convergence was measured by so called potential scale reduction and is reported in Table A3. The diagnostic results are reassuring, indicating that the simulation strategy yields samples from the actual posterior distributions of the considered probability variables $p^i$.

**Table A3. Diagnostic results for the MCMC simulation of probability variables corresponding to effectiveness and risk of non-serious adverse effects.**

| Probability variable | Intervention | Parameter | Potential scale reduction[a] |
|---|---|---|---|
| Effectiveness | High-dose methylprednisolone | $\alpha$ | 1.01 |
| Effectiveness | High-dose methylprednisolone | $\beta$ | 1.01 |
| Effectiveness | High-dose methylprednisolone | $p$ | 1.00 |
| Effectiveness | Low-dose methylprednisolone | $\alpha$ | 1.06 |
| Effectiveness | Low-dose methylprednisolone | $\beta$ | 1.07 |
| Effectiveness | Low-dose methylprednisolone | $p$ | 1.00 |
| Effectiveness | Placebo | $\alpha$ | 1.03 |
| Effectiveness | Placebo | $\beta$ | 1.03 |
| Effectiveness | Placebo | $p$ | 1.00 |
| Risk of non-serious adverse effects | High-dose methylprednisolone | $\alpha$ | 1.00 |
| Risk of non-serious adverse effects | High-dose methylprednisolone | $\beta$ | 1.00 |
| Risk of non-serious adverse effects | High-dose methylprednisolone | $p$ | 1.00 |
| Risk of non-serious adverse effects | Placebo | $\alpha$ | 1.03 |
| Risk of non-serious adverse effects | Placebo | $\beta$ | 1.03 |
| Risk of non-serious adverse effects | Placebo | $p$ | 1.00 |

[a] Values close to 1 are suggestive of a convergent simulation process.

*References*[*]


1. Gelman A, Carlin JB, Stern HS, Rubin DB. Bayesian Data Analysis. 2nd ed. Chapman & Hall / CRC; 2004.
2. Metropolis N, Rosenbluth AW, Rosenbluth MN, Teller AH, Teller E. Equation of state calculations by fast computing machines. J Chem Phys. 1953;21:1087-92.
3. Hastings WK. Monte carlo sampling methods using Markov chains and their applications. Biometrika. 1970;57:97-109.


[*] References number 1, 2, and 3 correspond to references number 23, 24, and 25, respectively, of the main article to which this additional file serves as supporting information.
