# Supplementary material for: Quantitative benefit-risk assessment of methylprednisolone in multiple sclerosis relapses
Source: BMC Neurol. 2015 Oct 16;15:206. doi: 10.1186/s12883-015-0450-x (PMC4609048; doi:10.1186/s12883-015-0450-x)
Supplement: Additional file 4: — Computation of upper risk limits for serious adverse effects based on individual case reports in VigiBase. (PDF 193 kb) [file 12883_2015_450_MOESM4_ESM.pdf]

Additional file 4: Computation of upper risk limits for serious adverse effects based on individual case reports in VigiBase®

Theory

A full account of this approach to risk quantification is provided by Caster *et al*. [1]. Below follows merely a brief overview, and interested readers are strongly encouraged to consult the original publication. It should be noted that although the approach generally provides both upper and lower risk limits, the focus of this additional documentation lies entirely on the former. The reason is that the lower limits require external information on the number of exposures, which unfortunately was unattainable in this assessment.

Underpinning the calculation of upper risk limits is a linking model between drug exposures and adverse events occurring in the real world, and events reported as suspected adverse reactions to a database. This model presupposes that any adverse event may be reported, but not more than once to the same database. It also presupposes that all reports describe adverse events that have actually occurred in real life. Whereas these presuppositions are unrealistic, they are generally not violated to such an extent that the validity of the upper risk limits is really threatened [1].

In this approach, risk is defined at the exposure level: it represents the fraction of exposures to a certain medicine that are followed by at least one event of the adverse effect under consideration. This definition fits precisely the decision-analytical framework for benefit-risk assessment applied in this study.

Now consider the reporting ratio constructed by dividing the number of reports on a medicine *X* together with an adverse effect *Y* by the total number of reports on *X*. Based on the linking model, one can show that this reporting ratio provides an upper limit for the risk (as just defined) of *Y* following *X*, if two assumptions are fulfilled: (i) the average number of adverse episodes* following exposure to *X* should be one or less; and (ii) the proportion of adverse episodes following *X* that are actually reported should be greater for episodes that contain *Y* than for episodes in general. The former assumption is more likely to hold the shorter the duration of treatment, and the healthier the patient population. The latter assumption should be generally valid for serious adverse effects.

It may be worthwhile pointing out that the wider the margins by which these assumptions are fulfilled, the more conservative the upper limits become. This also implies that any margin by which either of the assumptions is violated can be compensated for by a corresponding margin of fulfilment for the other assumption.

Application in this study

Here, methylprednisolone risk limits were computed not for adverse effects per se, but more specifically for serious adverse effect-outcome combinations. Some modifications to the general method have been applied, all outlined in the original publication [1]:

* An adverse episode is a set of adverse events that are temporally and clinically clustered so that they would be reported together, if at all reported.

- The targeted risk was the total risk excluding the background related to other drugs. In practice this means that those reports were excluded from the numerator count where methylprednisolone was listed only as a concomitant drug, and where there was any explicit information linking another drug to the adverse effect under consideration. Here, such implicating information was comprised by positive dechallenge and rechallenge reactions, and causality coded as ‘Certain’, ‘Probable’, or ‘Possible’.
- Based on clinical considerations, it was judged that methylprednisolone would be a very improbable cause of the included serious adverse effects if their onset was later than 180 days from start of treatment. Hence a restricted time to event onset of 180 days was employed, and those reports were excluded from the numerator count where it could not be inferred that the adverse effect under consideration started within this timeframe. The exception to this requirement was osteonecrosis, which is inherently difficult to diagnose.

Application of this approach requires careful consideration of the validity of the underlying assumptions. With respect to assumption (i) introduced above, it is clearly relevant that only short-term methylprednisolone treatment was considered in this study. However, assumption (i) could still be violated, since multiple sclerosis patients in acute relapse are far from being healthy. On the other hand, because of the high threshold for seriousness, assumption (ii) is likely to be fulfilled by a wide margin for a majority of the considered dose-adverse effect-outcome combinations. Because the two assumptions can compensate for each other, as noted above, the computed upper risk limits should generally be valid. The stark contrast between the high aggregate risk limits reported in Table 4 and the very low numbers of serious adverse events reported in clinical trials speaks in favour of this view. The obvious exceptions are those two adverse effect-outcome combinations that have zero reports for high-dose methylprednisolone (see Table 4 of the main article). For them, the proportion of reported adverse episodes is zero, which means that assumption (ii) cannot be fulfilled. As a pragmatic solution, those zero-valued limits were replaced by their corresponding upper risk limits for low-dose methylprednisolone.

References[†]

1. Caster O, Norén GN, Edwards IR. Computing limits on medicine risks based on collections of individual case reports. Theor Biol Med Model. 2014;11:15.

[†] Reference number 1 corresponds to reference number 28 of the main article to which this additional file serves as supporting information.
