# Supplementary material for: Quantitative benefit-risk assessment of methylprednisolone in multiple sclerosis relapses
Source: BMC Neurol. 2015 Oct 16;15:206. doi: 10.1186/s12883-015-0450-x (PMC4609048; doi:10.1186/s12883-015-0450-x)
Supplement: Additional file 5: — Sampling from qualitatively modelled utility variables in probabilistic decision analysis. (PDF 155 kb) [file 12883_2015_450_MOESM5_ESM.pdf]

Additional file 5: Sampling from qualitatively modelled utility variables in probabilistic decision analysis

This quantitative benefit-risk assessment employed a general framework of probabilistic decision analysis, which requires distributions over all constituent probability and utility variables. For the utilities, an approach was used whereby ab initio standard uniform distributions for all variables are modified according to a set of qualitative relations. Each relation expresses the comparative desirability of two considered clinical outcomes by declaring one of their corresponding utility variables greater than the other; for examples, please see Figure 6 of the main article. The below description is intended to provide an overview, and interested readers are referred to the original publication for details [1].

A permutation-based sampling technique

The modelling framework is flexible and does not require a relation to be specified between a given pair of utility variables. However, it is based on a core algorithm that does use a completely ordered set of dummy utility variables.

Assume that there are n utility variables that are all initially distributed as *Uniform*(0,1), and let $U_1,...,U_n$ denote the random variables that result from the specified relations. In other words, these are the random variables from which we wish to sample.

Further, let $V_1,...,V_n$ denote the random variables that result from a strict ordering $V_1 < ... < V_n$, where each V_j is also initially distributed as *Uniform*(0,1). Sampling from these dummy variables is straightforward with the aid of the $m \times n$ matrix X whose entries x_j^i are all *Uniform*(0,1) random numbers. Here, m is the total number of samples to be drawn, which in this assessment was 10,000, and i is the index for the current sample. It is now possible to sample from the dummy variables $V_1,...,V_n$ one at the time, starting with the largest [2]:

$$v_j^i = \begin{cases} \left(x_j^i\right)^{1/n} & \text{for } j = n \\ \left(x_j^i\right)^{1/j} v_{j+1}^i & \text{for } j = n-1, n-2, \ldots, 1 \end{cases} \tag{1}$$

Next it must be determined what permutations of the utility variables $U_1,...,U_n$ that are implied by the set of specified relations. Consider a simple example where $n = 4$ and the following has been specified: $U_4 < U_3$, $U_4 < U_2$, $U_3 < U_1$, and $U_2 < U_1$. Note that there is no relation specified between U_2 and U_3. While in this example it is clear that the only possible permutations are $U_4 < U_3 < U_2 < U_1$ and $U_4 < U_2 < U_3 < U_1$, the set of possible permutations must be algorithmically constructed in more complicated situations.

Regardless of how the set of permutations has been constructed, it is imperative to see that each permutation is equally probable. The reason is simply that all utility variables have the same ab initio distribution. In the example considered above, the probability is ½ for both possibilities $U_3 < U_2$ and $U_2 < U_3$, corresponding to the two respective permutations listed. Hence, for each sample iteration, one of the possible permutations is selected at random, and then the values sampled for the dummy variables are assigned accordingly. Again using the same small example, if in the i:th iteration the permutation $U_4 < U_3 < U_2 < U_1$ was selected, values would be assigned in the following way:

$$\begin{pmatrix} u_1^i \\ u_2^i \\ u_3^i \\ u_4^i \end{pmatrix} = \begin{pmatrix} v_1^i \\ v_2^i \\ v_3^i \\ v_4^i \end{pmatrix} \tag{2}$$

If instead the permutation $U_4 < U_2 < U_3 < U_1$ was selected, the value assignments would be the following:

$$\begin{pmatrix} u_1^i \\ u_2^i \\ u_3^i \\ u_4^i \end{pmatrix} = \begin{pmatrix} v_1^i \\ v_3^i \\ v_2^i \\ v_4^i \end{pmatrix} \tag{3}$$

In the long run, each permutation will be selected about the same number of times, and the values sampled for a given variable U_j will be distributed according to the position of that variable in the various possible permutations.

Accommodating minimum utility differences

The modelling framework also permits the inclusion of so called minimum utility differences, which in effect introduce extra separation between groups of utility variables. In this benefit-risk assessment, a minimum utility difference was introduced between lethal and non-lethal clinical outcomes. The value of this difference was one of the parameters altered in the sensitivity analysis. (For more details, see the Methods section of the main article.)

Here it will be assumed that the value d of the minimum utility difference is given. In this case, the dummy variables are related as $V_1 < ... < V_k < V_k+d < V_{k+1} < ... < V_n$. Clearly it must hold that $0 \leq d < 1$.

The algorithm presented in Equation 1 must now be adapted slightly. First, two parallel series of values s_j^i and t_j^i are sampled according to

$$s_j^i = \begin{cases} \left(x_j^i\right)^{1/n}(1-d) & \text{for } j = n \\ \left(x_j^i\right)^{1/j} s_{j+1}^i & \text{for } j = n-1, n-2, \ldots, 1 \end{cases} \tag{4}$$

and

$$t_j^i = \begin{cases} \left(x_j^i\right)^{1/n}(1-d)+d & \text{for } j = n \\ \left(x_j^i\right)^{1/j}\left(t_{j+1}^i - d\right)+d & \text{for } j = n-1, n-2, \ldots, 1 \end{cases} . \tag{5}$$

It is important to note that the same supporting matrix X is used in both Equation 4 and Equation 5. Sampled values for the dummy variables are then assigned in the following way:

$$v_j^i = \begin{cases} s_j^i & \text{for } j = 1, \ldots, k \\ t_j^i & \text{for } j = k+1, \ldots, n \end{cases} \tag{4}$$

Finally, values are assigned to the actual utility variables $U_1,...,U_n$ based on the sampled values v_j^i in exactly the same way as described above for the case without minimum utility differences. It should be noted that the inclusion of a minimum utility difference in itself implies that $U_p < U_q$ for all combinations of p in $\{1,...,k\}$ and q in $\{k+1,...,n\}$.

Although it is not made use of in the present benefit-risk assessment, the described framework naturally generalises to situations with more than one minimum utility difference.

References[*]

1. Caster O, Norén GN, Ekenberg L, Edwards IR. Quantitative benefit-risk assessment using only qualitative information on utilities. Med Decis Making. 2012;32:E1-E15.
2. Caster O, Ekenberg L. Combining second-order belief distributions with qualitative statements in decision analysis. In: Ermoliev Y, Makowski M, Marti K, editors. Managing Safety of Heterogeneous Systems (Lecture Notes in Economics and Mathematical Systems 658). Berlin Heidelberg: Springer-Verlag; 2012. p. 67-87.

[*] References number 1 and 2 correspond to references number 15 and 29, respectively, of the main article to which this additional file serves as supporting information.
