# Supplementary material for: Quantitative benefit-risk assessment of methylprednisolone in multiple sclerosis relapses
Source: BMC Neurol. 2015 Oct 16;15:206. doi: 10.1186/s12883-015-0450-x (PMC4609048; doi:10.1186/s12883-015-0450-x)
Supplement: Additional file 6: — Definitions of included serious adverse effects. (PDF 111 kb) [file 12883_2015_450_MOESM6_ESM.pdf]

Additional file 6: Definitions of included serious adverse effects

Each serious adverse effect included was defined as a set of MedDRA preferred terms, as reported in Table A6. For most of the adverse effects the starting point was one or several Standardised MedDRA Queries (SMQs), from which terms were chosen by the clinical expert (IRE) on the basis that they corresponded to the intended adverse effect. To reduce workload, the selection was made from the set of terms listed on any of the methylprednisolone reports assigned to either the low- or the high-dose group. This means that if a term belonging to the considered SMQ is not included here, it might not have been actively excluded; rather, it might not even have been considered.

Table A6. Definitions of included serious adverse effects, as sets of MedDRA preferred terms.

Adverse effect	SMQ used as basis[a]	Algorithm	MedDRA preferred term[a]	Term category[b]	Term implies life-threatening reaction[c]	Term implies persistent disability[c]
Acute severe allergy	Anaphylactic reaction (broad)	A or (B and C)	Anaphylactic reaction	A	Yes	No
			Anaphylactic shock	A	Yes	No
			Anaphylactoid reaction	A	Yes	No
			Bronchospasm	B	No	No
			Laryngeal oedema	B	No	No
			Laryngospasm	B	No	No
			Angioedema	C	No	No
			Face oedema	C	No	No
			Periorbital oedema	C	No	No
			Swelling face	C	No	No
			Urticaria	C	No	No

Diabetes	Hyperglycaemia / new onset diabetes (narrow)	NA	Blood glucose increased	NA	No	No
			Diabetes mellitus	NA	No	Yes
			Diabetes mellitus inadequate control	NA	No	No[d]
			Diabetic hyperglycaemic coma	NA	Yes	No[d]
			Diabetic ketoacidosis	NA	Yes	No[d]
			Glucose tolerance impaired	NA	No	No
			Glycosuria	NA	No	No
			Hyperglycaemia	NA	No	No
			Type 1 diabetes mellitus	NA	No	Yes
			Type 2 diabetes mellitus	NA	No	Yes
Cardio-pulmonary distress	Haemodynamic oedema, effusions and fluid overload (narrow)	NA	Acute pulmonary oedema	NA	Yes	No
			Pleural effusion	NA	No	No
			Pulmonary oedema	NA	Yes	No
	Acute central respiratory depression (broad)	NA	Acute respiratory distress syndrome	NA	Yes	No
			Acute respiratory failure	NA	Yes	No
			Apnoea	NA	Yes	No
			Cardiopulmonary failure	NA	Yes	No
			Cyanosis	NA	No	No
			Dyspnoea	NA	No	No
			Respiratory arrest	NA	Yes	No
			Respiratory depression	NA	No	No
			Respiratory distress	NA	No	No
			Respiratory failure	NA	Yes	No
Gastrointestinal haemorrhage	Gastrointestinal haemorrhage (narrow)	NA	Duodenal ulcer haemorrhage	NA	No	No
			Enterocolitis haemorrhagic	NA	No	No
			Gastric haemorrhage	NA	No	No
			Gastric ulcer haemorrhage	NA	No	No
			Gastritis haemorrhagic	NA	No	No
			Gastrointestinal haemorrhage	NA	No	No
			Gastrointestinal ulcer haemorrhage	NA	No	No
			Haematemesis	NA	Yes	No
			Haematochezia	NA	No	No
			Intestinal haemorrhage	NA	No	No
			Intra-abdominal haematoma	NA	No	No
			Melaena	NA	No	No
			Peptic ulcer haemorrhage	NA	No	No
			Rectal haemorrhage	NA	No	No
			Ulcer haemorrhage	NA	No	No
	Gastrointestinal perforation (narrow)	NA	Abdominal abscess	NA	No	No
			Duodenal ulcer perforation	NA	Yes	No
			Gastric perforation	NA	Yes	No
			Infectious peritonitis	NA	Yes	No
			Intestinal perforation	NA	Yes	No
			Peptic ulcer perforation	NA	Yes	No

Hepatotoxicity[e]	Hepatic failure, fibrosis and cirrhosis and other liver damage-related conditions (narrow)	NA	Ascites	NA	Yes	No
			Asterixis	NA	Yes	No
			Hepatic failure	NA	Yes	No
			Hepatic necrosis	NA	Yes	No
			Hepatic steatosis	NA	No	Yes
			Hepatocellular injury	NA	No	No
			Hepatotoxicity	NA	No	No
	Hepatitis, non-infectious (narrow)	NA	Hepatitis	NA	No	No
			Hepatitis acute	NA	No	No
			Hepatitis cholestatic	NA	No	No
			Hepatitis toxic	NA	No	No
	NA	NA	Blood bilirubin increased	NA	No	No
			Hepatic enzyme abnormal	NA	No	No
			Hepatic enzyme increased	NA	No	No
			Hypertransaminasaemia	NA	No	No
			Liver function test abnormal	NA	No	No
			Transaminases increased	NA	No	No
	NA	At least 2 of 3 terms required on same report	Alanine aminotransferase increased	NA	No	No
			Aspartate aminotransferase increased	NA	No	No
			Gamma-glutamyltransferase increased	NA	No	No
Osteonecrosis	NA	NA	Osteonecrosis	NA	No	Yes
Pancreatitis	Acute pancreatitis (broad)	A or (B and C)	Pancreatitis	A	No	No
			Pancreatitis acute	A	No	No
			Pancreatitis necrotising	A	Yes	No
			Blood amylase increased	B	No	No
			Lipase increased	B	No	No
			Abdominal pain	C	No	No
			Abdominal pain upper	C	No	No
			Abdominal tenderness	C	No	No
			Acute abdomen	C	No	No
			Ascites	C	No	No
			Fat necrosis	C	No	No
			Ileus paralytic	C	No	No
Ventricular arrhythmia and cardiac arrest	Shock-associated circulatory or cardiac conditions (excl torsade de pointes) (narrow)	NA	Acute left ventricular failure	NA	Yes	No
			Adams-Stokes syndrome	NA	Yes	No
			Cardiac arrest	NA	Yes	No
			Cardio-respiratory arrest	NA	Yes	No
			Circulatory collapse	NA	Yes	No
			Pulse absent	NA	Yes	No
			Shock	NA	Yes	No
			Ventricular fibrillation	NA	Yes	No
	Ventricular tachyarrhythmias (broad)	NA	Torsade de pointes	NA	No	No
			Ventricular arrhythmia	NA	No	No
			Ventricular extrasystoles	NA	No	No
			Ventricular fibrillation	NA	Yes	No
			Ventricular tachycardia	NA	No	No

Myopathy	Rhabdomyolysis / myopathy (broad)	NA	Blood creatine phosphokinase increased	NA	No	No
			Muscle necrosis	NA	No	Yes
			Myopathy	NA	No	Yes
			Rhabdomyolysis	NA	Yes	No
Seizures	Convulsions (narrow)	NA	Convulsion	NA	No	No
			Epilepsy	NA	No	No
			Grand mal convulsion	NA	No	No
			Status epilepticus	NA	Yes	No
Psychosis	Psychosis and psychotic disorders (narrow)	NA	Delusion	NA	No	No
			Hallucination	NA	No	No
			Hallucination, auditory	NA	No	No
			Hallucination, tactile	NA	No	No
			Hallucination, visual	NA	No	No
			Hallucinations, mixed	NA	No	No
			Illusion	NA	No	No
			Paranoia	NA	No	No
			Psychotic disorder	NA	No	No
			Schizophreniform disorder	NA	No	No

[a] The MedDRA version used was 14.1.

[b] If applicable, the term category is used to algorithmically determine whether the adverse effect of interest is on a given report.

[c] Life-threatening reactions and persistent disabilities are two of the possible serious outcomes considered in this benefit-risk assessment. Whether or not there is a term on a report that implies either of these outcomes for a certain adverse effect is one of the criteria considered when assigning serious outcomes to reported adverse effects; for a complete description, see Additional file 2.

[d] This term was interpreted as describing a reaction whereby the drug caused an acute and reversible worsening of a pre-existing condition; therefore it was not considered to intrinsically imply persistent diabetes as caused by the drug.

[e] Terms that relate to chronic liver injury have actively been excluded.
