# Supplementary material for: Quantitative benefit-risk assessment of methylprednisolone in multiple sclerosis relapses
Source: BMC Neurol. 2015 Oct 16;15:206. doi: 10.1186/s12883-015-0450-x (PMC4609048; doi:10.1186/s12883-015-0450-x)
Supplement: Additional file 7: — Process for selecting published studies used to estimate effectiveness and risk of non-serious adverse effects. (PDF 97 kb) [file 12883_2015_450_MOESM7_ESM.pdf]

## Additional file 7: Process for selecting published studies used to estimate effectiveness and risk of non-serious adverse effects

As described in the Methods sub-section ‘Estimation of probability variables’, estimation of effectiveness and risk of non-serious adverse effects was based on published studies included in, cited by, or citing any of the available systematic reviews on methylprednisolone in multiple sclerosis. Table A7 presents the contribution from each available source to the total pool of actually used studies. In the table, the first two eligibility criteria apply to both effectiveness and risk: to be further considered, any study would need to have at least one relevant treatment arm (i.e. high- or low-dose methylprednisolone or placebo for at most 31 days) and it would need to include patients in acute relapse and diagnosed with either relapsing-remitting or progressive multiple sclerosis. To be useful for estimation of effectiveness, the study would need to be randomised, double-blind, and report clinical results as the fraction of patients with an improvement of at least one EDSS point compared to start of treatment. To be useful for estimation of risk of non-serious adverse effects, the study would need to be prospective and report the number of patients with at least one non-serious adverse event.

**Table A7. Contribution from various sources to the pool of published studies used to estimate effectiveness and risk of non-serious adverse effects.**

| Source of studies[a] | Basis for considering studies further | Number of considered studies | Eligibility (number of studies)[b] | | | |
|---|---|---|---|---|---|---|
| | | | At least one relevant treatment arm | Relevant indication for use | Useful for estimation of effectiveness | Useful for estimation of risk of non-serious adverse effects |
| Meta-analysis by Miller et al. [2] | Studies included in source; see Table 2 in [2] | 6 | 6 | 6 | 6 | 4 |
| Meta-analysis by Brusaferri et al. [8] | Studies included in source; see Table 1 in [8] | 12 | 8 | 5 | 5 | 3 |
| Cochrane review by Burton et al. [9] | Studies included in source; see reference list of [9] | 5 | 4 | 4 | 2[c] | 2[c] |
| Cochrane review by Filippini et al. [10] | Studies included in source; see reference list of [10] | 6 | 5 | 5 | 4 | 3 |
| Cochrane review by Ciccone et al. [21] | Studies included in source; see reference list of [21] | 3 | 2 | 0 | 0 | 0 |
| Literature review by EFNS task force [1,22] | Preliminary inclusion based on abstracts of papers referenced in source | 32 | 31 | 20 | 9 | 9 |
| All papers citing any of the above systematic reviews[d] | Preliminary inclusion based on abstracts of papers referenced in source | 2 | 2 | 2 | 0 | 2[e] |
| **Total, including duplicates:** | | | | | **26** | **23** |
| **Total, unique:** | | | | | **10[f]** | **11[f,g]** |

[a] Reference numbers refer to the main article to which this table is supporting information.
[b] The indication for use was only considered among those studies where there was at least one relevant treatment arm. Similarly, the studies’ respective usefulness for estimation was only considered if the indication for use was relevant.
[c] One study was unpublished at the time of the Cochrane review. Its full results were published in October 2013 and are included in this assessment.
[d] According to SCOPUS as of 26th September 2013.
[e] For one of the studies, a more descriptive scientific publication was identified and used as replacement of the originally retrieved article.
[f] Six studies were considered useful for both effectiveness and risk of non-serious adverse effects.
[g] Only ten of the eleven studies were actually used: one study had to be excluded as the risk for low-dose methylprednisolone could not be estimated in the intended manner. (See the main article’s Methods sub-section ‘Estimation of probability variables’ for further details.)
